# Provider and patient perceptions on the usage of whole-body magnetic resonance imaging in oncology: a scoping review

**DOI:** 10.1186/s40644-026-01093-x

**Published:** 2026-07-23

**Authors:** Nazm Berry, Dow-Mu Koh, Anne Miles

**Affiliations:** 1 School of Psychological Sciences, Birkbeck University of London, London, UK; 2 Department of Radiology, Royal Marsden Hospital and Division of Radiotherapy and Imaging, Institute of Cancer Research, London, UK

**Keywords:** Adults, Magnetic resonance imaging, Whole body imaging, Oncology, Behaviour and behaviour mechanisms

## Abstract

Whole-body MRI (WB-MRI) is increasingly used for early diagnosis, cancer staging and treatment monitoring, but adoption remains sub-optimal. The aim of this scoping review was to map research on patient and provider perspectives of WB-MRI in oncology, and identify methodological limitations and evidence gaps. The review followed the JBI methodological framework and the PRISMA-ScR checklist. Five electronic databases were searched from inception to 29th March 2025: Cochrane Library, PubMed, APA PsycINFO, APA Psych Articles, and Academic Search Complete. Screening, data extraction, and quality assessment were performed by two authors independently, using COVIDENCE software, with results summarized via narrative synthesis. Twenty-eight studies met eligibility criteria, covering four distinct populations: general population (n = 8), people with cancer pre-disposition syndromes (n = 7), or suspected or confirmed cancer (n = 10), and radiologists (n = 3). Few studies examined beliefs about WB-MRI. Limited evidence shows awareness of WB-MRI attributes, such as the absence of ionising radiation, predicts preferences among cancer patients. Research on radiologists shows low levels of WB-MRI adoption in the USA and UK, attributed to limited scanner availability and a lack of trained radiologists. Among patients, uptake is modest (60%) in both population cohort studies and those recommended for surveillance, but the role of scan beliefs in predicting uptake has not been explored. Undergoing WB-MRI, or being notified of incidental findings, does not appear to cause adverse psychological consequences, but cancer specific concerns were not always assessed and research not always adequately powered. Evidence of provider and patient perspectives towards WB-MRI is limited and of varying quality. The development of validated measures examining WB-MRI beliefs and experience would enhance research and facilitate future data synthesis.

**Clinical trial number** Not applicable.

**Supplementary Information:**

The online version contains supplementary material available at https://doi.org/10.1186/s40644-026-01093-x.

## Background

Oncology has witnessed a paradigm shift with the advent of WB-MRI [1–3]. WB-MRI can detect lesions as small as 3–4 mm [4], often without the use of a contrast agent [5–7]. Furthermore, the absence of ionising radiation makes it particularly beneficial for people with cancer, or at high risk of developing the disease [8]. WB-MRI has greater sensitivity for the detection of bone lesions than both bone scintigraphy (BS) and 18-FDG PET/CT [3, 9–17], with comparable diagnostic accuracy to PET-CT/CT for both skeletal [18], and soft-tissue metastases [19, 20]. WB-MRI based surveillance results in improved survival among patients with germline TP53 mutations [21–23]. As a result, WB-MRI is recommended as the gold standard for detecting bone marrow involvement in myeloma [24], as a first-line imaging technique for evaluating patients with newly diagnosed myeloma and solitary plasmacytoma [25], and for surveillance in cancer predisposition syndromes [26, 27]. WB-MRI is also emerging as a method of cancer screening among asymptomatic members of the population [26, 28], but is not yet considered widely accessible or cost effective for routine clinical use.

Despite its advantages, the wider adoption of WB-MRI within clinical practice remains inconsistent internationally [29] due to the time and expertise required to process, read and interpret images [30–32] and the high cost, meaning few centres have access to WB-MRI [28]. Patients find WB-MRI challenging [33–35]. In addition, WB-MRI can lead to the discovery of incidental findings (IFs) - unanticipated discoveries that are not related to the objective of the exam but have potential health consequences for patients [36] and may result in adverse psychological consequences.

While previous systematic reviews have examined the comparative effectiveness of WB-MRI, to our knowledge, no reviews have explored patient and provider perspectives on WB-MRI. The aim of the current scoping review was, therefore, to map the existing literature on the knowledge, attitudes, behaviours, and preferences relating to WB-MRI adoption, as well as the psychological impact of undergoing WB-MRI in order to understand both provider and patient perspectives and identify gaps in the literature and areas for future research.

## Methods

The review was conducted in accordance with JBI methodological guidelines [37] and reported using the PRISMA-ScR checklist [38].

### Aim

This scoping review was conducted in the context of the National Institute of Health and Care Research (NIHR) - funded i4i Challenge study (NIHR202161), which aims to evaluate the clinical integration of WB-MRI for assessing bone disease in patients with advanced prostate cancer, using a CE-labelled computer software toolkit to help radiologists understand and interpret WB-MRI scans more accurately and efficiently.

### Protocol and registration

A protocol outlining the objectives, methods, and procedures of the current scoping review was submitted to the Open Science Framework (OSF) and can be accessed via the following link: https://osf.io/5htjf/.

### Eligibility criteria

The eligibility criteria were developed using the PICO framework (see Table 1).

**Table 1:** Eligibility criteria

Inclusion Criteria	Exclusion Criteria
*Population*	
· Healthy patients / asymptomatic patients (e.g. being offered WB-MRI as cancer screening) · People at higher risk of getting cancer · Cancer patients/survivors · All patients to be aged 18 or over · Health-care providers (specifically radiologists, radiographers, oncologists).	· Cancer patients aged under 18 · Exclude other health care professionals (e.g. nurses)
*Intervention/Exposure*	
· Use of WB-MRI · WB-MRI can be used before (screening), during or after diagnosis, and during or after cancer treatment (e.g. diagnosis, treatment monitoring, monitoring for recurrence).	· Not WB-MRI but MRI for specific location (e.g. breast, prostate). · Imaging modalities other than MRI (e.g. Pet-CT).
*Comparator/ Context*	
· Studies with or without a comparison group.	
*Outcome*	
· Primary outcomes: Knowledge, attitudes, beliefs, preferences or behaviour (use of) in relation to WB-MRI, including reasons for not using WB-MRI. · Secondary outcomes: Psychological burden, quality of life associated with use of WB-MRI or findings arising from WB-MRI.	· Clinical outcomes associated with WB-MRI (e.g. diagnostic accuracy studies).
*Study characteristics*	
· Any study design (e.g., randomized trials, non-randomized trials, pre-post studies, mixed-method studies, qualitative designs).	· Review studies · Protocol studies · Case studies · Commentaries · Conference abstracts · PhD theses · Grey literature · Language other than English

### Study selection and screening

References were exported into Covidence, where duplicates were removed. Title and abstract screening, and subsequent full text screening were done independently by two authors (AM and NB). Any disagreements were resolved via discussion, or input from the third author (DMK, a radiologist with experience in WB-MRI).

### Data extraction

The following information was extracted: author(s), year of publication, country where the study was conducted, aims/purpose, population, study design, sample size, sample age and gender, outcome measures (e.g. knowledge, attitudes/preferences, behaviours), and key findings.

### Critical appraisal of individual sources of evidence

Quality assessment of the included studies was conducted using established frameworks - for example the JBI template for qualitative studies, and quasi-experimental studies [39], and the National Institute of Health (NIH) quality assessment template for pre-post, observational, and case-control studies [40].

### Synthesis of results

The extracted data is presented in graphs and tables, in line with the objective of the scoping review. This is accompanied by a narrative summary of the results.

## Results

### Selection of sources of evidence

**Fig. 1:**
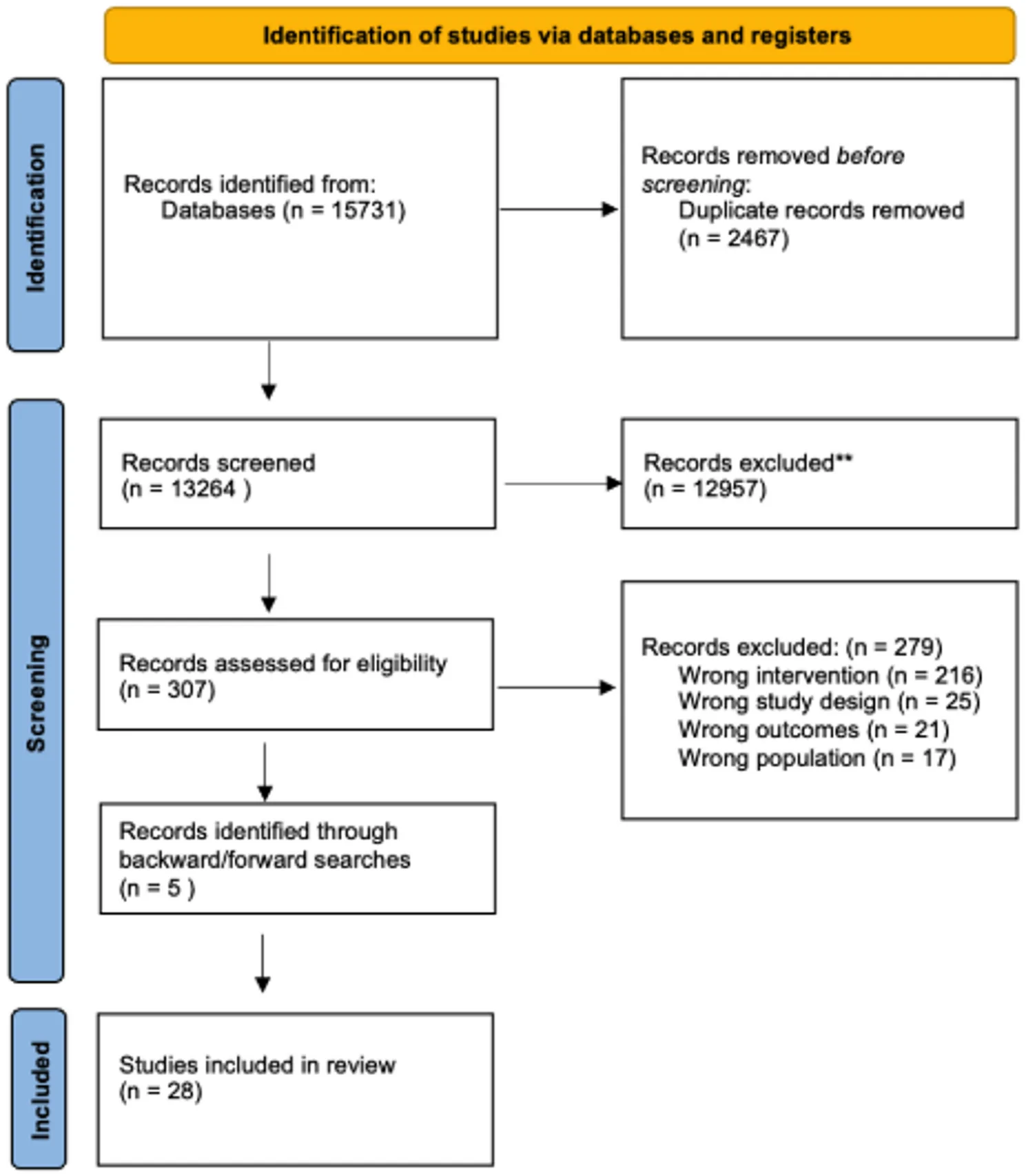
PRISMA flowchart

A PRISMA flowchart [41] showing the screening and selection process is presented in Fig. 1.

### Information sources and search strategy

The following databases were searched from inception to 29th March 2025: Cochrane Library, PubMed, APA PsycINFO, APA Psych Articles, and Academic Search Complete.

A search strategy was developed for each database, with help from the subject librarian at Birkbeck College, based on the key concepts: “WBMRI”; “Cancer”; “Knowledge, attitudes/beliefs, and behaviours”; and “clinicians and patients” (see Supplementary material Appendix 1 for the PubMed search string).

### Characteristics of sources of evidence

**Fig. 2:**
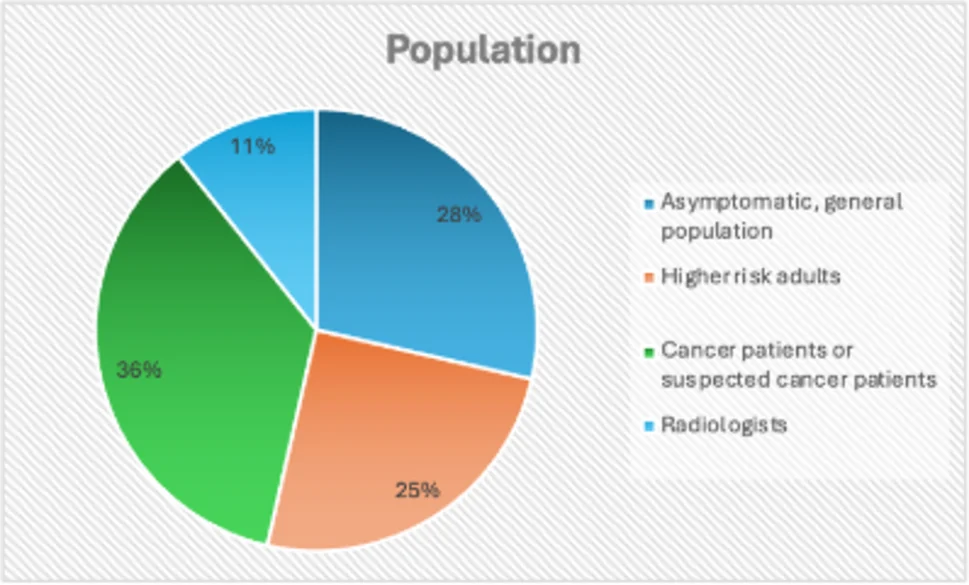
Population

**Fig. 3:**
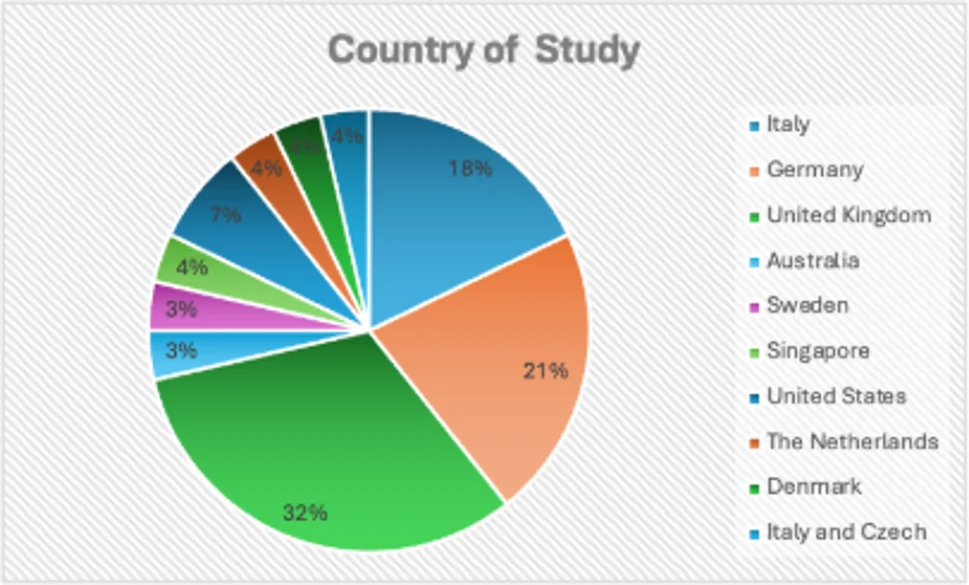
Country of study

**Fig. 4:**
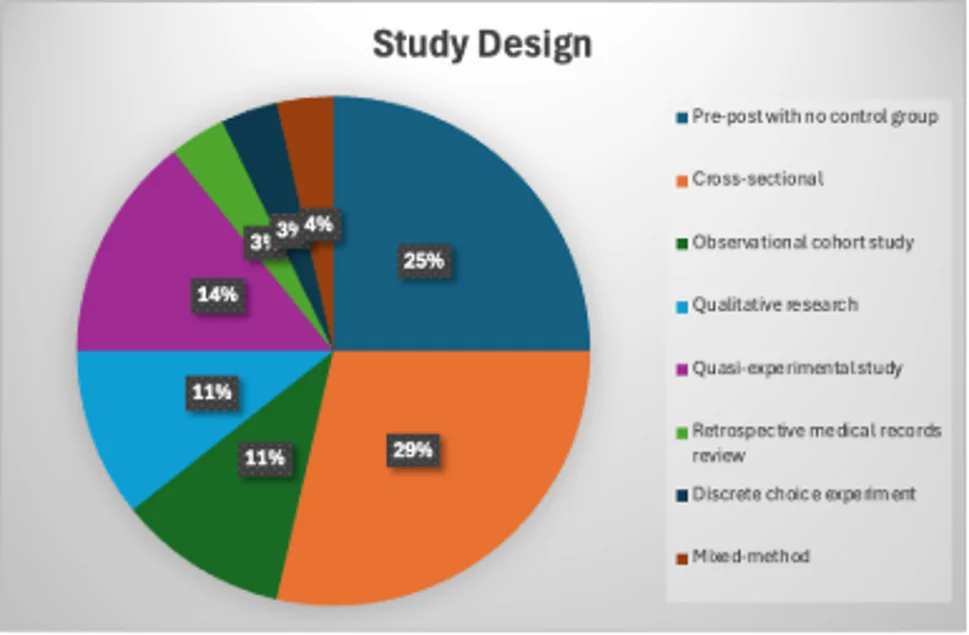
Study design

A graphical representation of the study populations, cancer type, country of study, and study designs can be found in Figs. 2, 3 and 4 and Fig. 1S in Supplementary material.

#### General population screening

Eight studies explored the use of WB-MRI for screening in asymptomatic members of the general population [29, 42–48]. Studies examined: knowledge, beliefs or expectations about WB-MRI [29, 42, 43]; reasons for WB-MRI screening participation [46, 47]; understanding, attitudes towards, or impact of IF disclosure [44, 46, 47]; scan experience, e.g. discomfort: [29, 42, 44]; scan satisfaction [42, 43, 47, 49]; scan acceptability [42]; and the psychological impact of undergoing WB-MRI [29, 45, 48], or being notified of incidental findings [29, 42, 44–48]. Sample sizes ranged from *n* = 65 to *n* = 4420.

#### Cancer predisposition syndromes

Seven papers examined the use of WB-MRI in the context of cancer predisposition syndromes [49–55]. Studies examined: knowledge of recommended surveillance guidelines [55], perceived personal cancer risk [50, 55], perceived efficacy of WB-MRI [49, 51, 52], perceived benefits and barriers to WB-MRI surveillance [49, 52, 53], uptake of surveillance [54, 55], participant willingness to have another WB-MRI [49, 50], scan experience [49, 51, 52], satisfaction with screening [50], and the psychological impact of undergoing WB-MRI-based surveillance [50, 51, 53, 55]. Sample sizes ranged from *n* = 13 to *n* = 88.

#### Cancer patients

Ten papers examined WB-MRI among people with suspected or confirmed cancer [33–35, 56–62]. Studies examined: patient knowledge or beliefs about WB-MRI [33, 60], scan preferences [57–61], provision of WB-MRI as part of first-line imaging in myeloma between 2016 and 2022 [62], scan experience [33–35, 56, 57, 61], and the psychological impact of undergoing WB-MRI [58]. Sample sizes ranged from *n* = 36 to *n* = 895.

#### Radiologists

Three studies were conducted on radiologists reported use of WB-MRI [63–65]. Studies examined WB-MRI practice patterns in general in 2018 [63], practice in relation to plasma cell malignancies/ myeloma in 2017 [64] and 2019 [65]. Sample sizes ranged from n = 61 to n = 164.

### Critical appraisal within sources of evidence

Studies assessing knowledge, attitudes and beliefs about WB-MRI, as well as scan expectations and experience, typically relied on non-validated measures (e.g. 33, 60), while studies assessing psychological impact and quality of life used validated ones (e.g. 45, 50). Studies involving higher-risk populations and cancer (or suspected cancer) patients often had smaller sample sizes than studies on the general population. Pre-post without a control group and observational/ cross-sectional studies were the most common designs (further details of quality appraisal are shown in the Appendices 2a and 2b).

### Synthesis of results

Study details of all included papers are shown in Table 2 and in Appendix 3 (Supplementary material).

**Table 2:** Summary of included studies (*n* = 28)

Author(s), year of publication and countries of study	Aim/ objective	Study design	Population	Sample size	Age (mean, sd or range); Gender
**General Population**					
Busacchio et al., 2021 (Italy)	To evaluate the satisfaction of asymptomatic subjects who self-referring Whole-Body Magnetic Resonance Imaging (WB-MRI) for early cancer diagnosis.	Pre-post with no control group	Asymptomatic, general population	65	Median 51 (30 to 73); 55.4% male
Busacchio et al., 2023 (Italy)	To identify the personal characteristics (sociodemographics, personality, expectations) that predict higher satisfaction with the WB-MRI examination, in a sample of asymptomatic individuals.	Pre-post with no control group	Asymptomatic, general population	154	52.8 (11.8); 57.8% male
Conti et al., 2025 (Italy)	To investigate the long-term psychological consequences of AF (abnormal findings) detection following WB-MRI for cancer screening in asymptomatic individuals.	Pre-post with no control group	Asymptomatic, general population in receipt of abnormal findings	121 (61 completed 4 year follow-up)	52.6 (11.4); 63% male
Hegedus et al., 2019 (Germany)	To evaluate the feasibility and validity of a follow-up questionnaire designed to assess participant feedback and incidental finding management in the whole-body MRI sub-study of the German National Cohort (GNC), using a single-centre pilot trial to inform necessary adjustments prior to large-scale implementation.	Observational cohort study (test-retest study so more specialised than this)	Asymptomatic, general population	86	58.7 (8.0); 44% male
Korbmacher-Böttcher et al., 2022 (Germany)	To analyze long-term psychosocial effects of MRI participation and IF disclosure within a population-based cohort	Quasi-experimental study	Asymptomatic, general population	855 (643 no MRI, 212 MRI)	62.4 (5.5) (no MRI) 62.4 (5.7) (MRI); 42% male (no MRI) 55% male (MRI)
Schlett et al., 2021 (Germany)	To assess the frequency, management and psychosocial consequences of incidental findings reporting in a population-based cohort study undergoing whole-body MR imaging.	Quasi-experimental study	Asymptomatic, general population	400 (243 completed both pre and post scan questionnaires)	56.3 (9.2); 58% male
Schmidt et al., 2013 (Germany)	To understand the attitudes, experiences and psychosocial consequences among participants who have been notified about incidental findings of clinical relevance after undergoing a wb-MRI examination.	Observational cohort study	Asymptomatic, general population in receipt of incidental findings	405	55.2 (12.2) (women); 61.6 (11.6) (men); 52.6% female
Schmidt et al., 2016 (Germany)	To examine the long-term impact of whole-body MRI and the disclosure of incidental findings on quality of life (QoL) and depressive symptoms in a general population cohort.	Quasi-experimental study	Asymptomatic, general population	4420 (2188 MRI, 1410 eligible but no MRI, 822 ineligible)	51.2 (14.1) (MRI); 53.6 (16.4) (eligible but no MRI) 51.2 (17.0) (non-eligible); 50.9% female (MRI); 52.8% female (eligible but no MRI); 50.9% female (ineligible)
**Cancer Predisposition**					
Bancroft et al., 2020 (United Kingdom)	To investigate the short-term and medium-term psychosocial effects, HRQoL and acceptability of WB-MRI scanning carried out as part of the SIGNIFY study.	Quasi-experimental study	Higher risk (TP53 carriers)	88 (44 carriers, 44 controls)	37.6 (19 to 58) (carriers); 38.3 (22 to 59) (controls); 61.3% female (carriers); 61.3% female (controls)
McBride et al., 2017 (Australia)	To assess the psychosocial impact in TP53 mutation carriers of WB-MRI screening as part of the Surveillance in Multi-Organ Cancer (SMOC+) protocol (first 12 months of participation in the trial), measuring their unmet needs, anxiety and depression levels as well as cancer worry using psychological questionnaires and in-depth interviews about their experiences of screening.	Mixed methods (Pre-post with no control group and qualitative)	Higher risk and cancer patients (TP53 carriers, or first degree relative with TP53)	17 (11 with past history of cancer, 6 unaffected by cancer); 9 completed interview	43.3 (21 to 65); 52.9% male
Neves et al., 2023 (United Kingdom)	To understand how people with A-T (ataxia–telangiectasia) and their parents feel about cancer surveillance using whole-body magnetic resonance imaging (MRI) to inform the future development of cancer surveillance guidelines.	Qualitative research	Higher risk (people with ataxia–telangiectasia) and their parents	13 (9 parents of children with A-T and 4 adults with A-T)	29 (6.6) (adults with A-T); not reported (parents of children with A-T); 100% female (adults with A-T); not reported (parents of children with A-T)
Omran et al., 2023 (Sweden)	To evaluate the psychosocial consequences of surveillance with WBMRI in individuals with the heritable TP53 related cancer (hTP53rc) syndrome with regard to cancer worry, perceived benefits and risks to surveillance and overall health.	Observational cohort study	Higher risk (TP53 carriers)	60	40 (18 to 74); 65% female
Que et al., 2025 (Singapore)	To evaluate the diagnostic performance of WB-MRI as a screening tool for Li-Fraumeni syndrome (LFS), constitutional mismatch repair deficiency syndrome, hereditary paraganglioma-pheochromocytoma syndrome (HPGL- PCC), and other CPS.	Retrospective medical records review	Higher risk (cancer predisposition syndromes)	59	Median 32 (1 to 74); 57.6% female
Rippinger et al., 2020 (Germany)	To investigate cancer surveillance habits and distress among adult pathogenic TP53 germline variant carriers in Germany.	Cross-sectional	Higher risk (TP53 carriers)	49	40.0 (12.6); 93.9% female
Ross et al., 2017 (United States)	To identify the psychosocial impact of LFS (Li-Fraumeni Syndrome) comprehensive screening options on individuals with LFS and determine whether psychosocial factors may influence screening adherence.	Qualitative research	Higher risk (TP53 carriers)	20	39.1 (18 to 61); 80% female
**Cancer Patients**					
Adams et al., 2014 (The Netherlands)	To assess and compare patient experience of whole-body magnetic resonance imaging (MRI) to that of computed tomography (CT) for staging newly diagnosed lymphoma	Cross-sectional	Cancer patients (lymphoma)	36 (32 adults, 4 children)	50.2 (19.4); 58.3% male
Dyrberg et al., 2018 (Denmark)	To investigate patient experience and acceptance regarding18F-sodium fluoride (NaF) positron emission tomography/computed tomography (PET/CT), 11 C-choline-PET/CT, whole-body magnetic resonance imaging (WB-MRI), and 99mTc-hydroxymethane diphosphonate (HDP) single photon emission/computed tomography (SPECT/CT).	Cross-sectional	Cancer patients (prostate cancer)	149	Median 72 (49 to 90); 100% male
Evans et al., 2017 (United Kingdom)	To describe the experience and acceptability of whole-body magnetic resonance imaging (WB-MRI) staging compared with standard scans among patients with highly suspected or known colorectal or lung cancer.	Qualitative research	Cancer patients or suspected cancer patients (lung and colorectal)	51 (26 colorectal, 25 lung)	64 (colorectal) 65 (lung) (40 to 89); 60.8% male
Evans et al., 2018 (United Kingdom)	To evaluate the perceived patient burden and acceptability of WB-MRI compared to standard staging investigations, and to identify predictors of reduced patient tolerance.	Pre-post with no control group	Cancer patients or suspected cancer patients (colorectal and lung)	115 (62 colorectal, 54 lung)	Median 66.3 (31 to 89); 58.3% male
Mazzocco et al., 2023 (Italy)	To assess anxiety and potential causes of anxiety in cancer patients undergoing whole-body magnetic resonance imaging (WB-MRI)	Pre-post with no control group	Cancer patients or suspected cancer patients (breast, prostate)	70 (46 breast; 24 prostate)	60.3 (37 to 82); 65.7% female
Miles et al., 2019 (United Kingdom)	To determine the importance placed by patients on attributes associated with whole-body MRI (WB-MRI) and standard cancer staging pathways and ascertain drivers of preference.	Discrete choice experiment	Cancer patients or suspected cancer patients (colorectal and lung)	138 (66 colorectal, 72 lung)	64.7 (10.9); 60.9% male
Miles et al., 2020 (United Kingdom)	To identify predictors of patient preferences for either WB-MRI or CT/ PET-CT when staging known or highly suspected colorectal or lung cancer.	Pre-post with no control group	Cancer patients or suspected cancer patients (colorectal and lung)	107	64.7 (11.9); 57.9% male
Oliveri et al., 2018 (Italy)	To investigate patients’ acceptance of WB-MRI as a procedure for cancer staging and follow up.	Pre-post with no control group	Cancer patients (mixed)	135	55.4 (13.4); 57% female
Pinto et al., 2024 (Italy and Czech)	To report a secondary outcome of the prospective international multicenter Imaging Study in Advanced ovArian Cancer (ISAAC Study), detailing patients’ experience with abdomino-pelvic ultrasound, whole-body contrast-enhanced computed tomography (CT), and whole-body diffusion-weighted magnetic resonance imaging (WB-DWI/MRI) for pre-operative ovarian cancer work-up.	Cross-sectional	Cancer patients or suspected cancer patients (ovarian)	144	59.9 (14.6); 100% female
Quinn et al., 2024 (United Kingdom)	To evaluate the provision of imaging at diagnosis of myeloma from the service user perspective with a specific focus on how the experiences of patients align with the National Institute for Health and Care Excellence (NICE) guidelines (NG35, 2016) on first-line imaging practice for myeloma in the United Kingdom.	Cross-sectional	Cancer patients and carers (myeloma)	895	30 or under (1.8%); 41–50 (9.1%); 51–60 (25.1%); 61–70 (32.8%); 71–80 (26.4%); 81 years or older (3.4%); 53.3% female
**Radiologists**					
Feldhaus et al., 2020 (United States)	To evaluate musculoskeletal (MSK) radiologist whole-body magnetic resonance imaging (WBMRI) practice patterns in an effort to better understand current MSK clinical utilization and the need for standardized coding.	Cross-sectional	Society of Skeletal Radiology (SSR) members	164	50% (academic practice); 37.2% (private practice); 12.8% (hybrid private practice with academic partnership)
Westerland et al., 2018 (United Kingdom)	(1) To evaluate compliance of current UK imaging practice with reference to National Institute for Health and Care Excellence best-practice clinical guidelines for plasma cell malignancies. (2) To identify factors which may influence diagnostic imaging choices.	Cross-sectional	Clinical haematologists and radiologists with an interest in plasma cell malignancies	61	50.8% (district general hospitals); 45.9% (teaching hospitals); 3.3% (unconfirmed sites)
Westerland et al., 2021 (United Kingdom)	To evaluate current national imaging practice in myeloma with reference to National Institute for Health and Care Excellence (NICE) guidelines (NG35, 2016) and compare results with an initial survey conducted in 2017 (61 participating sites).	Cross-sectional	UK radiology departments treating myeloma patients and with a Royal College of Radiologists (RCR) Audit Lead	114	68% (district general hospitals); 19% (teaching hospitals); 13% (tertiary referral centres)

### General population screening

#### Primary outcomes

Most participants in population-based cohort studies underwent WB-MRI to know if they were healthy (86–97%) but supporting science was also a common driver (77–91%) [46, 47]. Hegedus et al. found 95% of participants considered IF notification very important, with 46% saying they would not have participated without it [44]. However, preferences for disclosure varied: 90–91% agreed to the reporting of IFs requiring urgent treatment, and 73% wanted to know about IFs indicating suspected conditions, where treatment was not necessary but possible [47]. Almost half wanted to know about any findings, regardless of clinical relevance [44].

People’s understanding of the clinical significance of their IFs also varied. While Hegedus et al. reported perfect agreement between the study co-ordinator and participant understanding, Schmidt et al. found poor correspondence, with patient and radiologist agreement that findings were life threatening observed in only 42% of cases, resulting in an agreement level close to zero (kappa = 0.02) [47]. Prior knowledge of IFs was mixed, with 53.6% having no prior knowledge, but knowledge level strongly predicted people’s understanding of their notification letter: only 3.7% with full prior knowledge failed to understand their letter, compared to 9.5% with partial knowledge and 25.6% with no prior knowledge (*p* < 0.001) [47].

Willingness to have another WB-MRI was high among people who had already undergone one [44], with two studies reporting 95% would be willing to have a WB-MRI again [42, 44]. However, uptake among people in the general population was much lower. One population-based cohort study reported an uptake rate of only 60%, with those attending for WB-MRI healthier than those who did not, reflecting a healthy cohort effect.

#### Secondary outcomes

Some level of scan discomfort was common, for example, 56% reported some discomfort during WB-MRI, with moderate and moderately severe discomfort reported in 14% and 5% respectively, with exam duration and claustrophobia the most common causes [29]. However average levels of scan discomfort were low (below 2 on a 5 point scale) [43]. While discomfort does not appear to differ by previous WB-MRI experience, actual discomfort experienced has been shown to be better than expected, both overall (*p* = 0.0007), and specifically for noise (*p* = 0.01), shortness of breath (*p* = 0.003) and claustrophobia (*p* = 0.0007), although not exam duration [42].

WB-MRI scan satisfaction was high, with rates of high satisfaction/ contentment varying from 83 to 96% [29, 42, 47], but was unrelated to previous experience of WB-MRI [42]. Higher scan satisfaction was associated with higher perceived scan usefulness (β = 0.245, *p* = 0.002) [43]. However, the proportion reporting high satisfaction decreased significantly with an increased number of IFs (*p* = 0.05), although the number of IFs did not appear to raise patient concerns following the scan [42].

Population based cohort studies suggest that undergoing WB-MRI screening has minimal adverse psychological consequences [45, 48]. For example, in their study of 4420 members of the general population, Schmidt et al. found no significant group-by-time interactions when comparing WB-MRI participants and non-participants on changes in quality of life or depression before vs. after the scan [48]. Most studies also found no evidence that receipt of incidental or abnormal findings resulted in adverse psychological effects [45, 46, 48]. The exception was one study, which found distress was higher among those with a subjective interpretation of the IF as life threatening (*p* = 0.01), although the measure of distress used was not validated [47].

### Cancer predisposition syndromes

#### Primary outcomes

Few studies have examined knowledge about recommended TP53 germline variants–specific surveillance. One cross-sectional survey found most knew what was recommended (78%), with 18% unsure, and 4% uninformed [55]. In addition, they found 77% adhered to the surveillance recommended following their diagnosis, but 23% were non-adherent − 19% attended less often than recommended and 4% had stopped participating. Adherence was significantly more likely among people who expressed higher satisfaction with genetic testing and counselling (*p* = 0.019) but was unrelated to demographics, cancer history, or level of TP53 germline variant–related distress.

Quantitative research into beliefs about surveillance is also limited. One survey study showed that those undergoing surveillance reported high perceived benefits and low perceived barriers to WB-MRI screening [53] and qualitative research has demonstrated the perceived benefits of screening include improved cancer outcomes [49, 51, 52], the absence of ionising radiation, the ability of WB-MRI to detect non cancer-related problems [52], and providing peace of mind [49]. Barriers to WB-MRI surveillance included logistical issues, the biggest one being insurance coverage for WB-MRI [49]. In their survey study, Rippinger et al. found that only 57% reported their insurance companies fully covered the costs of surveillance, although 31% of participants were unable to provide any information on the coverage they had. Reasons for non-adherence to surveillance also included frequent confrontation with the condition, or the belief that further adherence would not be necessary after prophylactic mastectomy [55].

Consistent with findings from general population screening, willingness to have a WB-MRI among people who had already had one was relatively high at 77–98% [50], but was lower in a more representative sample: a retrospective medical records review of patients who had been recommended WB-MRI surveillance found an uptake rate of 57% [56].

#### Secondary outcomes

TP53 mutation carriers reported more cancer worry [50, 53], and more frequent, and clinically significant, intrusive and avoidant thoughts about cancer compared to age and sex-matched controls [50]. For example, the proportion with clinically significant intrusive thoughts about cancer prior to getting the results of their WB-MRI was 21 vs. 5% in carriers vs. controls respectively (*p* < 0.0005), and 31 vs. 2% at one year follow-up (*p* < 0.0005) [50]. However, there is no evidence at present that WB-MRI screening exacerbates cancer worry, as no significant increase has been observed from baseline to one year after WB-MRI scan completion [50, 51]. However, while cancer intrusion scores declined significantly over time, Bancroft et al. found cancer worry and cancer avoidance, while unchanged among carriers, declined significantly in the control group, raising the possibility that WB-MRI may not be as reassuring among carriers. However, the group-by-time interaction was not formally tested [50]. There is also no evidence WB-MRI based surveillance affects anxiety, depression or quality of life [50].

In addition, there is currently no evidence that undergoing WB-MRI has an adverse effect on either people with a history of cancer [50, 53] or those requiring further investigations following WB-MRI [50]. For example, Bancroft et al. found no difference in carriers by personal cancer history in anxiety, depression, cancer worry or avoidant thoughts about cancer. While intrusive cancer thoughts declined significantly pre-results to 12 weeks follow-up in carriers without a personal history of cancer but not those with, the interaction between group and time was not formally tested. Similarly, Omran et al. found no differences in cancer worry by previous cancer history, except on one of the 8 cancer worry items, which actually decreased more among people with a previous cancer history than those without (*p* = 0.014) [53].

Qualitative studies have highlighted the psychological burden that surveillance can confer, beyond the scan itself, via the need for frequent follow-up visits, the discovery of findings requiring further investigation, reminding people of their increased cancer risk, as well as increased cancer burden in the family [51, 52, 55]. However, positive consequences were also noted, with some participants saying surveillance screening helped them cope with their increased risk status [51], and satisfaction with screening was high [50].

### Cancer patients

#### Primary outcomes

Few studies have examined WB-MRI knowledge and beliefs among people with suspected or confirmed cancer. Only one study examined awareness of one of the key benefits of WB-MRI (the absence of ionising radiation), finding low awareness (42%) [60].

Three studies found WB-MRI was preferred to PET-CT/CT by a majority, ranging from 52 to 77% [58–60]. However, two studies found it was the least popular compared to ultrasound or CT, with only 13% preferring WB-MRI [61] or the scan patients would most like to avoid when compared to NaF-PET/CT and choline PET/CT, with 47% saying they would avoid WB-MRI if given the choice [57]. Reasons favouring WB-MRI included awareness that WB-MRI does not involve radiation [60], reduced time to diagnosis and/or the need for fewer scans in total [59]. For example, one study found more positive beliefs about the benefits of WB-MRI were the only significant predictor of patient preference for WB-MRI (*p* = 0.047), controlling for other factors including demographic variables, comorbidities, emotional distress and scan burden:, 64.4% of people who were aware that WB-MRI did not use radiation preferred WB-MRI to CT/PET-CT, compared to 43.5% of people unaware of this attribute [60]. Reasons against WB-MRI included scan discomfort [57].

Only one study examined WB-MRI in the context of myeloma, finding the provision of WB-MRI as part of first-line imaging in myeloma had remained unchanged between 2016 and 2022, despite the introduction of guidelines in 2016 recommending WB-MRI [62].

#### Secondary outcomes

Two studies reported superior patient experience with WB-MRI compared to other scans [33, 56]. For example, Adams et al. found patients reported significantly less worry before WB-MRI than CT (1.47 vs. 1.83, scale 1–4, *p* = 0.007), experienced WB-MRI as significantly less unpleasant (1.49 vs. 1.83, scale 1–5, *p* = 0.01) and felt significantly better shortly after WB-MRI compared to CT (1.64 vs. 1.93, scale 1–4, *p* = 0.003). However, mean scores for worry and unpleasantness were low and scores for post-scan wellbeing indicate high levels, with differences attributed to the necessary preparations before CT scanning (i.e. insertion of intravenous line, ingestion of contrast medium), not required for whole-body MRI in staging of lymphoma [56].

Four studies reported inferior experience with WB-MRI [34, 35, 57, 61], mainly due to discomfort related to enclosed space, noise and scan duration [34, 35, 57, 61]. For example, Evans et al. found mean scan burden scores for WB-MRI were significantly greater than those of CT/PET-CT (2.21 vs. 1.81, *p* < 0.001), with WB-MRI conferring significantly greater feelings of claustrophobia and discomfort related to noise than CT/PET-CT (mean scores for claustrophobia WB-MRI vs. CT: 2.81 vs. 1.51; *p* < 0.001; WB-MRI vs. PET-CT: 3.04 vs. 1.98; *p* < 0.001; mean scores for noise: WB-MRI vs. CT: 2.84 vs. 1.73; *p* < 0.001; WB-MRI vs. PET-CT: 2.85 vs. 1.63; *p* < 0.001). Patients with higher psychological distress, existing comorbidities and pulmonary symptoms also reported greater scan burden [34, 35]. For example, Evans et al. found in adjusted analyses, that higher psychological distress (β = 0.223; *p* = 0.025) and presence of comorbidities (β = 0.191, *p* = 0.048) significantly predicted scan burden, while demographic variables and cancer type (lung vs. colorectal) did not.

Factors improving WB-MRI tolerance included levels of information received prior to the scan [33], good staff communication before and during the scan [34, 57], and coping strategies employed during the scan (e.g. distraction, breathing techniques, focusing on health benefit) [34]. Only one study examined the psychological impact of undergoing WB-MRI, finding no difference between pre vs. post WB-MRI anxiety scores, but participants had not received their results when post WB-MRI anxiety was assessed [58].

### Radiologists

#### Use of WB-MRI

Studies on the use of WB-MRI among radiologists show low levels of adoption. For example, in the USA only 32% of Society of Skeletal Radiology (SSR) members reported that their institutions routinely performed WB-MRI [63], although the response rate to the survey was very low at 11%. In the UK, challenges to implementing a WB-MRI service included scanner availability (52%), financial constraints (49%), reporting time (46%), and lack of trained radiologists to report (46%), with only 48% of responding hospitals reporting onsite WB-MRI availability [65].

## Discussion

This scoping review mapped the existing literature on patient and provider perspectives of WB-MRI in oncology. Twenty-eight studies were identified covering four distinct populations: asymptomatic members of the general population (*n* = 8); people with cancer predisposition syndromes (*n* = 7); people with suspected or confirmed cancer (*n* = 10); and radiologists (*n* = 3).

### Summary of evidence

#### Knowledge, attitudes, and uptake

The evidence available indicates that adoption of WB-MRI remains suboptimal among both providers and patients (e.g. 54, 65). Among radiologists, challenges to implementing a WB-MRI service include scanner availability, financial constraints, reporting time, and lack of trained radiologists to report [64, 65], with onsite availability of WB-MRI noted in only 48% of UK hospitals [65]. Although willingness to have another WB-MRI appears very high among people who have already had one (e.g. 42, 44, 49, 50), uptake among more representative samples is modest, with studies showing approximately 60% of both the general population, and those recommended WB-MRI surveillance, take up the offer of a WB-MRI scan [48, 54].

Few studies have examined awareness of key WB-MRI attributes. One study showed that only 42% of cancer patients were aware WB-MRI did not use ionising radiation, but did not use a validated scale to assess patient knowledge. Some research has also shown that WB-MRI attributes, such as the absence of ionising radiation [60], fewer scans and a more rapid diagnosis [59] predict cancer patient preferences for WB-MRI over other staging pathways e.g. those including CT/PET-CT. In addition, beliefs in the usefulness of WB-MRI for diagnostic purposes have also been associated with higher scan satisfaction scores [43], and been shown to facilitate coping with the challenges of undergoing the scan [34], suggesting perceptions of WB-MRI may impact patient preferences and experiences, but there is a lack of studies and validated measures to draw any firm conclusions.

IF notification is viewed as an important benefit of WB-MRI [e.g [47, 52], but patient understanding of IFs is variable with one study showing very poor agreement between radiologists and patients regarding which IFs were classified as life threatening [47].

#### Scan experience and psychological impact

There is currently no evidence that either undergoing WB-MRI or being notified of IFs has adverse psychological consequences (e.g. 45, 48, 51). However, this conclusion must be interpreted with caution as studies on people with cancer predisposition syndromes used small samples and hence were often underpowered to detect significant effects. Furthermore, interactions between groups and changes in psychological outcomes over time were not always formally tested. Research on larger scale general population samples did not employ cancer-specific measures, such as cancer worry. This may result in adverse consequences being missed. For example, Bancroft et al. observed differences between people with cancer-predisposition syndromes and controls on cancer-specific measures but not measures of anxiety or depression [50]. Moreover, previous research has shown the psychological impact of medical test results, such as false positive results at cancer screening, is detected using cancer-specific rather than generic measures [e.g [66].

Scan experience was mixed, with claustrophobia, scan duration, and the need to lie still the most commonly reported sources of discomfort across different populations [34, 42, 49, 51, 52]. Additional challenges among people predisposed to cancer included the need for frequent follow-up visits, and constant reminders of cancer risk status [51, 55]. Numerous studies examined overall scan satisfaction or acceptability, but again did not use validated measures (e.g. 42–44, 61).

### Study designs and limitations

The majority of studies employed cross-sectional or pre-post designs without a control group, rather than stronger designs such as case-control studies or quasi-experimental designs. A number of studies, particularly on people with cancer or cancer predisposition syndromes, were based on small samples, often missing power calculations. Studies assessing psychological impact generally used validated measures, however a consistent methodological limitation across the studies was the reliance on non-validated measures to assess scan beliefs, experience and satisfaction. This limits the strength of conclusions that can be drawn and, critically, prevents meaningful data synthesis across studies. In addition, it is assumed that WB-MRI is a uniform technology, however, in practice different centres may utilise different protocols/scan set-up/examination times, which may affect participant experience and feedback across studies.

### Implications

The above limitations mean that the current evidence base should be regarded as preliminary, and findings interpreted with caution. Due to the heterogeneity of outcomes measures, study designs and the timing of measurements, a systematic review with the potential for meta-analysis is not warranted at this time. Research on people with cancer predisposition syndromes represents the most developed strand of the literature, with the greatest consistency in population characteristics and outcomes measured using validated scales, and would be a potential candidate for a future systematic review, particularly if more studies were conducted using a control group. Furthermore, the development and adoption of validated measures assessing WB-MRI-specific knowledge, beliefs and scan experience would facilitate future attempts to understand predictors of uptake and scan experience, and enable future data synthesis. The use of cancer-specific measures e.g. cancer worry, would also help strengthen the evidence base concerning the potential psychological impact of undergoing WB-MRI.

### Limitations of the review

The primary aim of the review was to explore knowledge, beliefs, attitudes, and uptake of WB-MRI. Databases using MeSH terms offered more comprehensive coverage of psychological outcomes. While forward and backward searches identified additional studies, it is possible that relevant papers were missed from databases not using MeSH terms. Furthermore, the search was limited to five databases, did not include any grey literature sources or conference abstracts, and was restricted to English-language papers. Finally, the context of our review was the use of a CE-labelled computer software toolkit to facilitate the interpretation of WB-MRI scans in advanced prostate cancer, so we restricted our population of health-care providers to those involved in WB-MRI acquisition and evaluation of treatment response to bone disease. Studies involving other healthcare professionals (for example surgeons, urologists, and nurses) were excluded. However, none of the articles captured in our search that were subsequently excluded on the basis of being the wrong population (e.g. urologists) were on WB-MRI but were on regional MRI (e.g. mpMRI prostate).

## Conclusions

Adoption of WB-MRI remains suboptimal both among providers and patients. The development of validated measures of knowledge about the characteristics of WB-MRI would enhance research into predictors of WB-MRI uptake and scan experience, and enable data synthesis to take place at a future date.

## Supplementary Information

Below is the link to the electronic supplementary material.

Supplementary Material 1

Supplementary Material 2

## Data Availability

The datasets used and/or analysed during the current study are available from the corresponding author on reasonable request.

## Abbreviations

18-FDG PET: 18F-fluorodeoxyglucose positron emission tomography
CE-labelled: Conformité européenne labelled
CT: Computed tomography
IF: Incidental findings
JBI: Joanna briggs institute
PET-CT: Positron emission tomography–computed tomography
PRISMA-ScR: Preferred reporting items for systematic reviews and meta-analyses extension for scoping reviews
WB-MRI: Whole body magnetic resonance imaging
