# Supplementary material for: Provider and patient perceptions on the usage of whole-body magnetic resonance imaging in oncology: a scoping review
Source: Cancer Imaging. 2026 Jul 23;26:144. doi: 10.1186/s40644-026-01093-x (PMC13679176; doi:10.1186/s40644-026-01093-x)
Supplement: Supplementary file 2 [file 40644_2026_1093_MOESM2_ESM.docx]

**SUPPLEMENTARY MATERIAL**

**Figure S1: Cancer type for cancer population**

**Appendix 1 : Search string**

(("whole body imaging"[MeSH Terms] OR "magnetic resonance imaging"[MeSH Terms] OR "whole body imaging"[Text Word] OR "magnetic resonance imaging"[Text Word] OR "whole body mri"[Text Word] OR "WBMRI"[Text Word] OR "WB-MRI"[Text Word] OR "MRI"[Text Word]) AND ("neoplasms"[MeSH Terms] OR "neoplasm*"[Text Word] OR "cancer"[Text Word] OR "oncology"[Text Word]) AND ("Behavior and Behavior Mechanisms"[MeSH Terms] OR "attitude*"[Text Word] OR "knowledge"[Text Word] OR "Anxiety"[Text Word] OR "barrier*"[Text Word] OR "belief*"[All Fields] OR "Behavior and Behavior Mechanisms"[Text Word] OR "uptake"[Text Word] OR "behav*"[Text Word] OR "preference*"[Text Word]) AND ("radiologists"[MeSH Terms] OR "oncologists"[MeSH Terms] OR "radiologist*"[Text Word] OR "radiographer*"[Text Word] OR "oncologist*"[Text Word] OR "patient*"[Text Word] OR "healthy subject*"[Text Word]) AND ("humans"[MeSH Terms] AND "english"[Language]) AND ("adult"[MeSH Terms] OR "adult"[MeSH Terms:noexp] OR ("middle aged"[MeSH Terms] OR "aged"[MeSH Terms]) OR "middle aged"[MeSH Terms] OR "aged"[MeSH Terms] OR "aged, 80 and over"[MeSH Terms] OR "young adult"[MeSH Terms])) AND (alladult[Filter] OR youngadult[Filter] OR adult[Filter] OR middleagedaged[Filter] OR middleaged[Filter] OR aged[Filter] OR 80andover[Filter]))

(NB: the term “behaviour and behavioural mechanisms” which encompasses terms such as “psychology”, “emotions”, “outcome expectancies”, “trust”, “patient satisfaction”, and “patient acceptance of healthcare.”).

**Appendix 2: Quality assessment**

For studies involving the general population, two studies were assessed using the NIH tool for observational studies (44, 47); three using the JBI tool for quasi-experimental studies (45, 46, 48); and three using the NIH tool for pre-post studies without a control group (29, 42, 43). For studies comprising a high-risk/cancer-predisposition population, the NIH tool for observational studies was used for three studies (53-55); the JBI tool for qualitative studies was used for two studies (49, 52); the NIH tool for pre-post studies without control group was used for one study (51); and the JBI tool for quasi-experimental studies for one study (50). Among studies involving cancer or suspected cancer patients, four studies were assessed using the NIH tool for observational studies (56, 57, 61, 62); four were assessed using the NIH tool for pre-post studies without a control group (33, 35, 58, 60); one using the JBI tool for qualitative studies (34); and one using the discrete choice experiment tool (59). Finally, among studies involving the radiologist population, all three studies were assessed using the NIH tool for observational studies (63-65).

***Appendix 2a: NIH tool for observational studies***

The NIH tool for observational studies was used for papers that adopted a cross-sectional design (55-57, 61-65), observational cohort study design (44, 47, 53); and one paper that used the retrospective medical records review (54). Across all 12 studies, sample size justifications were rarely provided, and some studies did not report whether the participation rate reached 50%. Furthermore, while outcome measures used were often clearly defined, they were either unvalidated or included a mixture of validated and non-validated measures. While some studies measured potential confounding variables and included appropriate statistical adjustments, the majority did not.

| **NIH TOOL : OBSERVATIONAL STUDY** | | | | | | | | | | | | | | |
| --- | --- | --- | --- | --- | --- | --- | --- | --- | --- | --- | --- | --- | --- | --- |
| **Study (Population)** | **Quality Assessment** | | | | | | | | | | | | | |
|  | Objective clearly stated? | Study population clearly specified? | Participation rate of eligible persons at least 50%? | Subjects recruited from the same populations with pre-specified inclusion and exclusion criteria? | Sample size justification provided? | Exposures of interest measured prior to the outcomes? | Timeframe sufficient to see an association between exposure and outcome? | Different levels of the exposure as related to the outcome? | Exposure measures clearly defined, valid, reliable, and implemented consistently? | Exposure(s) assessed more than once over time? | Outcome measures clearly defined, valid, reliable, and implemented consistently? | Outcome assessors blinded to the exposure status of participants? | Loss to follow-up 20% or less? | Key potential confounding variables measured and adjusted for? |
| **General population screening** |  |  |  |  |  |  |  |  |  |  |  |  |  |  |
| Hegedus et al., 2019 | Yes | Yes | Yes | Yes | No | Yes | Yes | No | Yes | No | Other | No | NR | No |
| Schmidt et al., 2013 | Yes | Yes | Yes | Yes | No | Yes | Yes | Yes | Yes | No | Other | Other | Yes | No |
| **Cancer pre-disposition syndromes** |  |  |  |  |  |  |  |  |  |  |  |  |  |  |
| Omran et al., 2023 | Yes | Yes | NR | Yes | No | Yes | Yes | No | Other | Yes | Other | No | Yes | No |
| Rippinger et al., 2020 | Yes | Yes | Yes | Yes | No | No | No | No | Yes | No | Other | Other | N/A | No |
| Que et al., 2025 | Yes | Yes | Yes | Yes | No | No | Other | Other | Other | Other | Yes | Other | N/A | No |
| **Cancer patients** |  |  |  |  |  |  |  |  |  |  |  |  |  |  |
| Adams et al., 2014 | Yes | Yes | Other | Yes | No | Yes | Yes | No | Yes | No | Other | No | Yes | No |
| Dyrberg et al., 2018 | Yes | Yes | Yes | Yes | No | Yes | Yes | Other | Yes | No | Other | No | Other | Yes |
| Pinto et al., 2024 | Yes | Yes | Yes | Yes | No | Yes | Yes | N/A | Yes | No | Other | NR | N/A | No |
| Quinn et al., 2024 | Yes | Yes | NR | Yes | Yes | No | N/A | N/A | Yes | No | Other | NR | N/A | No |
| **Radiologists** |  |  |  |  |  |  |  |  |  |  |  |  |  |  |
| Feldhaus et al., 2020 | Yes | Yes | No | Yes | No | No | N/A | Yes | No | No | Other | No | N/A | No |
| Westerland et al., 2018 | Yes | Yes | NR | Yes | No | No | N/A | No | Yes | No | Other | Other | N/A | No |
| Westerland et al., 2021 | Yes | Yes | Yes | Yes | No | No | N/A | Other | Yes | NR | Other | N/A | NR | No |

**Appendix 2b: NIH tool pre-post studies with no control group**

All eight studies with this design provided clear research objectives and well defined eligibility criteria, although details about whether all those who were eligible were enrolled in the study were not reported. In addition, while authors clearly defined their outcome measures, often providing the original questionnaire, they were typically unvalidated.

| **NIH TOOL : PRE-POST WITH NO CONTROL GROUP** | | | | | | | | | | | | |
| --- | --- | --- | --- | --- | --- | --- | --- | --- | --- | --- | --- | --- |
| **Study (Population)** | **Quality Assessment** | | | | | | | | | | | |
|  | Study question clearly stated? | Eligibility criteria clearly described? | Study participants representative of those who would be eligible for the intervention in the general or clinical population of interest? | All eligible participants that met the prespecified entry criteria enrolled? | Sample size sufficiently large to provide confidence in the findings? | Test/service/intervention clearly described and delivered consistently? | Outcome measures prespecified, clearly defined, valid, reliable, and assessed consistently? | People assessing the outcomes blinded to the participants' exposures/interventions? | Loss to follow-up 20% or less; was it accounted for in the analysis? | Did the statistical methods examine changes in outcome measures from before to after the intervention and report the P values? | Outcome measures taken multiple times before and after the intervention? | Did the statistical analysis take into account the use of individual-level data to determine effects at the group level? |
| **General population screening** |  |  |  |  |  |  |  |  |  |  |  |  |
| Busacchio et al., 2020 | Yes | Yes | Yes | No | Yes | Yes | Other | No | Yes | Yes | No | Other |
| Busacchio et al., 2023 | Yes | Yes | Yes | NR | Yes | Yes | Other | Other | Yes | N/A | No | NR |
| Conti et al., 2025 | Yes | Yes | Yes | NR | Yes | Yes | Other | NR | Yes | Yes | Yes | N/A |
| **Cancer pre-disposition syndromes** |  |  |  |  |  |  |  |  |  |  |  |  |
| McBride et al., 2017 | Yes | Yes | Yes | No | Yes | Yes | Yes | N/R | No | Yes | Yes | Other |
| **Cancer patients** |  |  |  |  |  |  |  |  |  |  |  |  |
| Evans et al., 2018 | Yes | Yes | Yes | No | Yes | Yes | Other | No | Yes | Yes | No | No |
| Mazzocco et al., 2023 | Yes | Yes | Yes | NR | Yes | Yes | Yes | N/A | NR | Yes | No | N/A |
| Miles et al., 2020 | Yes | Yes | Yes | No | Yes | Yes | Other | No | No | Other | No | No |
| Oliveri et al., 2018 | Yes | Yes | Yes | NR | Yes | Yes | Other | N/A | Yes | Yes | No | Other |

**Appendix 3: Summary of included studies (n= 28)**

| **Author(s), year of publication and countries of study** | **Methodological details** | **Findings** |
| --- | --- | --- |
| **General Population** |  |  |
| Busacchio et al., 2021 (Italy) | Before the scan: prior experience with MRI and WB-MRI; perceived personal risk of disease (diabetes, tumour, heart attack, HIV, meningitis); scan concern, perceived usefulness, perceived riskiness of scan; expected scan discomfort from noise, claustrophobia, shortness of breath, and scan duration; perceived level of personal knowledge of WB-MRI. After the scan (and meeting with radiologist to discuss findings including possible IFs): scan concern, perceived usefulness, perceived riskiness of scan; actual experience of WB-MRI with respect to noise, claustrophobia, shortness of breath, scan duration, level of perceived vulnerability; additional perceived knowledge of WB-MRI; scan satisfaction; acceptability compared to other scans; willingness to have another WB-MRI. General acceptability score consisted of perceived usefulness of WB-MRI, perceived risk related to WB-MRI, and general expected discomfort. | **Knowledge, attitudes and beliefs:** 44.6% participants felt they had a good or very good level of information/ knowledge about WB-MRI before the scan. 41.5% reported receiving more or much more additional information after the scan, with the proportion 73% vs 50% in people who had not previous had a WB-MRI compared to those who had (ns). Prior to WB-MRI, 98.5% rated perceived usefulness of WB-MRI "for your diagnostic process" to be high or very high. 89.2% did not view WB-MRI as "risky" either before or after the scan. 29% of subjects expected discomfort of some form with claustrophobia (27.7%) and exam duration (24.6%) the most common concerns (numbers not reported for all categories). **Incidental findings:** IFs were all oncological not suspicious findings or findings not relevant for the main purpose of the exam (cancer screening).  **Scan experience:** the majority did not experience discomfort due to noise (over 75%), claustrophobia (approx. 70%) shortness of breath (over 70%), exam duration (over 60%) (specific figures not reported but presented in figures). Discomfort experienced was better than expected in general (average across four sources of discomfort) (p=0.0007), and specifically for noise (p=0.01), shortness of breath (p=0.003) and claustrophobia (p=0.0007), but not exam duration. Discomfort did not differ by previous WB-MRI experience (not clear if this relates to reported general discomfort or difference between experienced vs expected). Higher levels of perceived personal risk of disease (p=0.03) and lower education (p=0.01) were significantly associated with a greater reduction between expected and experienced shortness of breath. Women reported experiencing less claustrophobia than expected compared to men (p value not reported). **Scan satisfaction:** 83.1% reported high levels of satisfaction with WB-MRI and this did not differ by previous experience of WB-MRI. The proportion reporting high satisfaction decreased significantly with the number of incidental findings (p=0.05), but levels of post-examination concern were not significantly associated with the number of incidental findings. **Scan acceptability:** Among people who had had other exams, 48% consider WB-MRI to be much more acceptable than other scan (alternative scans not reported). General scan acceptability scores increased significantly from pre to post WB-MRI (p=0.0001). 95.4% said they would be willing to have a WB-MRI again. |
| Busacchio et al., 2023 (Italy) | Before the scan: Personality traits (BIF-10); expectations about WB-MRI (expected usefulness, risks, noise, lack of air, and duration). After scan: evaluation of the WB-MRI procedure, including satisfaction with the procedure (specific items not given) | **Knowledge, attitudes and beliefs:** Perceived usefulness was high (mean score 4.35) while perceptions of anticipated scan risk and discomfort were low (means: risk=1.35, noise=1.40, lack of air=1.49, and duration=1.61, where expected discomfort 1 =no, 2=low, 3=moderate, 4=high, and 5=extreme). **Scan satisfaction:** Mean satisfaction scores were high (4.48). In unadjusted analysis, higher scan satisfaction was significantly correlated with higher agreeableness (r=0.180, p<0.05), higher perceived scan usefulness (r=0.211, p<0.01), lower perceived scan risk (r=-0.198, p<0.05), lower anticipated discomfort from scan duration (r=-0.166, p<0.05), and lower anticipated discomfort from lack of air (r=-0.289, p<0.001). No significant associations were found between scan satisfaction and anticipated discomfort from scan noise or demographic variables (age, gender or education). In adjusted analysis (with demographic, personality and expectations all entered together), higher agreeableness (beta=0.150, p=0.049), higher perceived scan usefulness (beta=0.245, p=0.002), and higher expected discomfort from noise (beta=0.172, p=0.036) significantly predicted higher satisfaction with WB-MRI, while higher expected discomfort from lack of air (beta=-0.303, p<0.001) had a negative impact on satisfaction. |
| Conti et al., 2025 (Italy) | Before the scan with a psychologist: Confidence, concerns and perceived scan utility, personality (BFI-10), and perceived illness severity and personal cancer risk - both absolute and comparative, demographics. After the scan with a psychologist after discussing scan results with the radiologist: discomfort and scan satisfaction, quality of life (SF-12), anxiety (HAM-A), depression (PHQ-9). | **Knowledge, attitudes and beliefs:** Most participants reported having no or only mild concern before the scan (59% and 40% respectively). Data on confidence and perceived scan utility were not reported. **Abnormal findings (AFs):** All participants had AFs, which were classified following ONCO-RADS guidelines (as defined by Petralia et al 2021): Category 1 (normal); Category 2 (benign findings highly likely) considered at low risk of cancer and were not suggested specific follow-up; Category 3 (benign finding likely) were considered at intermediate risk of cancer and performed further clinical examinations according to the anatomical region of the AF. No participants were classified in categories 4 or 5 (malignant findings likely/highly likely). **Psychological impact:** T0= just after the WB-MRI scan, T1= 1 year after the scan, T2= 4 years after the scan in a subsample. There was a significant increase in depression from T0 to T1 (T0=3.26, T1=3.35, p=0.007) but the change across all three time points was not significant. There were no significant interactions between any of the following and changes in anxiety, or QoL over time: number of AFs (low vs high); presence of suspicious findings (category 3 vs categories 1 or 2) or personal perceived risk of getting cancer. The personality dimension of conscientiousness was significantly associated with “depression progression” (direction not stated) (T0-T1, p=0.006; T0-T1-T2, p=0.045), and openness to experience with “anxiety progression” (direction not stated) over time (T0-T1, p=0.036, T0-T1-T2, ns). None of the other interactions between personality type and anxiety, depression or QoL were significant. **Scan experience:** 44% reported no discomfort during the scan, with 36% reporting mild, 14% moderate and 5% moderately severe discomfort. **Scan satisfaction:** 90% participants reported being very satisfied with the scan, 10% reported moderate satisfaction. |
| Hegedus et al., 2019 (Germany) | After the scan: all participants - motivation for participating in the MRI examination, clarity of pre-exam information, perceptions of scan duration, perceived stress during wait for IF notification, satisfaction with scan information, willingness to undergo another WB-MRI, demographics. Among those with an IF: whether the disclosed finding was previously known, whether the language of disclosure was clear enough to understand, degree to which they were distressed by the notification, how IF was followed up (if and which specialist was consulted, what kind of work-up was done, any complications during work-up, time until diagnosis and exact diagnosis). | Motivation for scan participation not reported. **Incidental findings - knowledge, attitudes and beliefs:** Participants all informed only IFs that were clinically relevant or life-threatening would be disclosed. 70% unaware of this IF communication principle. 95% rated receipt of IFs as very important. 46% stated they would not have taken part without IF notification. Almost half (number not reported) wanted to be informed about all findings, regardless of clinical relevance. Only 15% preferred to be informed only about medical conditions that were treatable. 97% rated IF reports as “well or very well to understand”. **Incidental findings - psychological impact:** The emotional stress while waiting for a result letter was rated severe by 7% and mild by 34%. 55% agreed IF disclosure had had a positive influence on their health status; 81% of all disclosed IFs were not previously known to the participant. Perfect agreement was achieved between the IF reporting as documented by the study coordinator and the participants’ reply in the survey (100%). **Scan experience:** More than 95% perceived scan duration, as not too long and hence tolerable. Satisfaction with scan information: Ninety-two percent were satisfied with the information given prior to the WB-MRI scan. Willingness to have again: About 95% said they would consider participating in another WB-MRI scan. |
| Korbmacher-Böttcher et al., 2022 (Germany) | Psychosocial and health assessments were conducted across three time points: exam 1 (KORA F4, six years before MRI), exam 2 (KORA-FF4, during MRI in 2013/2014), and exam 3 (KORA FIT, four years after MRI). The Depression and Exhaustion Scale (DEEX), the Somatization Scale, the Patient Health Questionnaire (PHQ-9), the Perceived Stress Scale (PSS-10), and the Short Form Health Survey (SF-12). DEEX, PHQ-9, and the Somatization Scale were assessed at all three exams, SF-12 at exams 1 and 2, and PSS-10 only at exam 3. Demographic and health-related variables (age, sex, BMI, family status, smoking, diabetes, hypertension, antidepressant use, and physical activity) were collected at every exam. | **Psychological impact:** Compared to MRI participants, non-participants had a higher psychosocial burden: higher PHQ-9 (3.3 vs 2.5, p<0.001); and lower MCS in the SF-12 (50.6 vs. 52.4, p=0.01) at exam 1; higher DEEX (8.6 vs. 7.7, p=0.01), PSS-10 (14.7 vs 13.7, p=0.03), and Somatisation (5.9 vs 4.8, p=0.001) at Exam 3. There was no change in quality of life among WB-MRI participants, and although there was a statistically significant increase in depression over four years in this group, changes were small and remained below clinical thresholds (2.5 to 3.1, p=0.02) , and differences at follow-up did not differ between recipients and non-recipients of WB-MRI. There was a significant increase in DEEX in the control group over time (7.9 to 8.6, p<0.001), and significant increases in somatisation in the WB-MRI group (4.2 to 4.8, p=0.013), the control group (4.7 to 5.9, p<0.001) and the WB-MRI group with no IFs (4.1 to 4.7, p=0.02). However, interactions between WB-MRI (yes/no) and time were not formally tested. **Incidental findings - psychological impact:** There were no significant differences in psychosocial outcomes among MRI participants with or without IF at any of the three time points. Looking at all three groups (no MRI, MRI with IFs, MRI without IFs) and adjusting for group differences in demographic and health-related variables, MRI with no IF had better psychosocial outcomes on PHQ-9, DEEX and somatisation than non-MRI participants, but the differences between MRI with IF and non-MRI were not significant. |
| Schlett et al., 2021 (Germany) | Before the scan: motivation for participation, perceived importance of IF notification (both life threatening and non-life-threatening), anticipated stress while waiting for the IF report, depression (PHQ-9). After the scan: motivation for participation, perceived importance of IF notification, actual stress felt while waiting for the IF report, preference for method of IF notification, depression (PHQ-9). | **Reasons for participation in WB-MRI:** Pre scan the most common reason was to “contribute to a scientific purpose” (91%), followed by “knowing whether I’m healthy” (86%), and “I have health complaints and would like to figure out the reason” (21%). Post scan there was a significant decrease in “contribute to a scientific purpose” (to 82%, p=0.01). The proportion citing the other reasons did not change significantly but “knowing whether I’m healthy” was the most common reason post-scan at 88%. (18% post-scan, ns). **Incidental findings - knowledge, attitudes and beliefs:** 56.8 % indicated that reporting IF in general would be “very important” for them pre-scan, which significantly decreased to 42.0 % after the scan (p<0.001). This decrease did not differ between those with and without IFs. 53% of participants stated they would have participated if absolutely no IFs would have been disclosed; this portion did not change significantly over time. The desire for IF reporting increased significantly over time (pre- vs. 6months-post-scan), for life threatening needing immediate action (91 vs 98%, p=0.002); not life threatening but treatment necessary 94% to 97% (ns) not life threatening, treatment not necessary but possible (72 % vs. 84 %, p = 0.001). Preference for communication of IF was by letter (77%), by personal appointment (12%). **Incidental findings - psychological impact:** a small portion of participants (3.4%) reported that waiting for the IF reporting added “definitely” or “very probably” to additional stress burden. There was no significant difference between the amount of added stress people expected compared to that experienced. Of participants with reported IF, 9 % felt that the results were “very burdening”, while 57 % classified the results as “very helpful”. Rates of moderate/ moderately severe depression did not differ between participants with and without reported IF. Rates overall were 3.3% for moderate (10-14 points) and 1.2% for severe depression (15 points or over). |
| Schmidt et al., 2013 (Germany) | After the scan: Reason for participation; distress while waiting for the result; satisfaction with the scan; the informed consent process and the reporting of findings and preferences regarding the future reporting of findings; novelty of disclosed findings; psychological consequences due to disclosed findings; satisfaction with WB-MRI. | **Reasons for participation in WB-MRI** were: “I will know whether I am healthy” (97.3%), “I wanted to support science” (76.8%), “I have some kind of physical complaint and want to learn more about the reason” (44.7%), “I no longer need to participate in a routine screening examination” (14.6%), “financial compensation” (4.4%). **Incidental findings - knowledge, attitudes and beliefs:** Only findings requiring further medical attention were eligible for disclosure. Just over half had IFs reported that they had previously been unaware of (53.6%), 25.9% had partial knowledge and 13.3% had full knowledge. Those with prior knowledge of the IF were more likely to understand the letter they were sent about the findings; only 3.7% with full prior knowledge did not understand the letter, compared to 9.5% with partial knowledge and 25.6% with no prior knowledge (p<0.001). There was poor correspondence between patient and radiologist classification of findings, with a kappa coefficient close to zero (k=0.02): 41.9% of findings patients believed were life threatening were actually classified as such by radiologists. The majority (90.4 %) agreed to the reporting of incidental findings in case of a life threatening disease that required immediate treatment, as well as in case of other diseases requiring urgent treatment (90.9 %). 72.8 % desired disclosure in case of suspected medical conditions not requiring medical treatment but where treatment was possible. Most (75.3 %) stated that they wanted to be informed about the absence of findings as well. The majority were content with a letter disclosing incidental findings (56.5 %) but one third would have preferred face-to-face talks with radiologists (37.5 %). **Incidental findings - psychological impact:** More than half (53.1 %) reported no distress while waiting for a potential notification of IFs. 9.9 % reported moderate to severe distress (as defined by the highest three distress categories). There was no significant relationship between distress and waiting time but a significantly higher proportion of females than males reported distress while waiting (14.1% vs. 5.2%, p=0.01). 66.2 % perceived the communication of incidental findings to be moderately or very helpful, but perceived helpfulness was not significantly associated with subjective severity of IFs. Psychological consequences were higher compared to social consequences. 6.9 % reported moderate to severe social impairment, whereas 28.6 % stated moderate to severe psychological distress. Distress (indexed by concerns about heath, difficulty sleeping, unrest, uncertainty, depressed mood) was higher among those with a subjective interpretation of the finding as life threatening (p=0.01, OR not reported). Compared to the period before MRI, 71.4 % felt “safer within their own body”. Scan satisfaction: The majority (96.0 %) were very content with their participation in the WBMRI examination. |
| Schmidt et al., 2016 (Germany) | Before and after the scan: Quality of life (SF-12) and depression (PHQ-9). | **Uptake:** Out of 4420 participants invited, 3598 were eligible, and 2188 underwent a WB-MRI, an uptake rate of 60.1%. **Psychological impact:** The observation time between baseline examination and survey was 2.47 years. There was no effect of WB-MRI participation among eligible participants on quality of life or depression. Difference between participants vs. non-participants - unstandardized beta (with 95% CIs): PCS = -0.06 (-0.30 to 0.18); MCS = -0.01 (-0.29 to 0.29); PHQ-12 = 0.08 (-0.03 to 0.18). Across all MRI-eligible participants there was a slight decrease in QoL scores over time but not depression. There were no significant group by time interactions for QoL or depression between MRI participants and non-participants. **Incidental findings - psychological impact:** Only findings requiring medical attention were disclosed. Approximately one third of MRI participants were notified about an incidental finding. There was no difference between those found to have IF and those not for any of the outcomes (main effect of WB-MRI and MRI x time interactions not significant), and none of the interactions between site of incidental finding and change in QoL or depression over time were significant. Difference between IF vs no IF - unstandardized beta (with 95% CIs): PCS = -0.03 (-0.39 to 0.33); MCS = -0.26 (-0.65 to 0.13); PHQ-12 = 0.03 (-0.10 to 0.15). |
| **Cancer Predisposition** |  |  |
| Bancroft et al., 2020 (United Kingdom) | TP53 carriers and age and sex-matched controls. Questionnaire booklets were administered at seven time-points: (1) study enrolment (baseline), (2) on arrival at the MRI appointment, (3) pre-MRI and (4) post-MRI results, and (5) 12, (6) 26 and (7) 52 weeks post results. Time points 5–7 were only administered to patients not diagnosed with cancer as a result of the WB-MRI. Measures: anxiety and depression (HADS), cancer worry (CWS-R), physical and mental health (SF36-II), perceived risk of cancer (compared with average person and personal lifetime risk), satisfaction with the process of screening (SSQ), distress caused by screening (HQ), the presence of intrusive thoughts relating to MRI screening and cancer (IES), state anxiety related to the MRI (STAI-B), acceptability of screening (would they take up the offer of another MRI scan next year, would they encourage a family member to attend for an MRI). | Outcomes relate to WB-MRI. **Psychological impact** - *group differences carriers vs. controls:* Carriers reported higher cancer worry at baseline, 26 week and 52 week follow-up (p<0.0005), higher levels of perceived risk for cancer at every time point (p<0.0005), and greater frequency, and more clinically significant intrusive and avoidant thoughts about cancer compared to the controls at every time point (p values from <0.01 to <0.005) (Proportion clinically significant intrusive thoughts carriers vs. controls before results: 21 vs. 5%, p<0.0005; at 52 week FU: 13 vs 2%, p<0.0005. Proportion clinically significant avoidant thoughts carriers vs. controls before results: 23 vs. 14%, p=0.036; at 52 week FU: 15 vs 4%, p<0.0005.). Carriers reported more frequent intrusive thoughts about MRI at 26 and 52 weeks (p values 0.05 to 0.02). Carriers reported poorer physical and mental health functioning than controls, but the effect size was small and means were close to population averages suggesting these differences were not clinically meaningful (e.g. carriers vs control baseline physical health: 49.2 vs. 52.1, p<0.007, 52 week FU 47.9 vs. 51.7, p=0.03; mental health baseline: 50.2 vs 54.0, p=0.03, 52 week FU not significantly different). There were no differences between carriers and controls in terms of prevalence of clinically significant anxiety and depression at any of the time points, or levels of state anxiety at the time of the MRI. There was little evidence of differences between the groups in terms of distress (stress-sensitive behaviours), as scores did not significantly differ over time in either group and significantly higher scores were only observed in carriers compared to controls at 12 week FU and not at any of the other time points. *Time differences carriers vs. controls:* Cancer intrusion scores declined over time in both groups (from pre-results to either 12 or 26 weeks FU, p values not reported). There was no significant change in cancer worry or cancer avoidance over time in carriers, but both declined significantly in controls between baseline and 52 weeks, however the interaction between group and time was not formally tested. No significant changes were observed in quality of life (either physical or mental functioning), distress over time, or avoidance or intrusive thoughts in relation to MRI in either carrier or controls. *Carriers with vs. without a personal history of cancer:* no differences between the two groups found for anxiety, depression, cancer worry or avoidant thoughts about cancer at any time point between those with vs. those without a family history of cancer. Intrusive cancer thoughts declined significantly pre-results to 12 weeks FU in carriers without a personal history of cancer but not in carriers with a personal cancer history, however the interaction between time and group was not formally tested. *Carriers requiring vs. not requiring further investigations:* no differences between the two groups were found for anxiety, depression, cancer worry, cancer-related intrusion or avoidance scores. **Scan satisfaction:** Satisfaction with screening was high and only differed between groups on one of six items (physical surroundings) with no differences in general satisfaction (carriers vs. controls 6.8 vs 7.3 - scale 5-25, low scores good). **Scan acceptability:** Acceptability of screening was high and did not differ with respect to encouraging a family member to attend but carriers were significantly more likely to agree to attend an MRI next year compared to controls (98% vs. 77%, p=0.015). Additional results: Some carriers substantially underestimated their cancer risk. 54-58% correctly perceived their cancer risk as strongly increased compared to the general population. |
| McBride et al., 2017 (Australia) | Questionnaires completed at baseline and 2, 12, 26 and 52 weeks post WB-MRI. Anxiety and depression (HADS) assessed at all time points, cancer worry (CWS) assessed at all time points except 2 weeks post MRI, intrusive thoughts in relation to cancer and in relation to MRI assessed at all time points except baseline. Qualitative: hopes for screening, awareness of cancer risk, experience of WB-MRI surveillance. | **Psychological impact:** Outcomes relate to WB-MRI. Significant reduction in anxiety scores from baseline to two weeks post WB-MRI (decrease of 1.2 points from baseline to 2 weeks post WB-MRI, p=0.025), but no significant change in depression. No significant change in cancer worry over time using linear mixed modelling (baseline to 1 year FU). No significant change in intrusive thoughts about cancer during FU. Intrusive thoughts about MRI were very low for all participants and formal analysis was therefore not done. *Qualitative findings:* Four qualitative themes were reported: perceived efficacy of screening (WB-MRI viewed as effective at early detection, despite being told absence of evidence for this currently in this population); burden of screening (scan burden, scan findings requiring further investigation, reminder of cancer risk) burden of a TP53 germline mutation (cancer in the family, inability to share cancer worry, guilt of transmission to children); containment of the psychological impacts of carrying a TP53 germline mutation (access to screening and doctors helped cope with increased risk). |
| Neves et al., 2023 (United Kingdom) | A-T patients' and their parents' perceptions regarding WBMRI. Questions focused on the potential impact of a cancer screening program on families, current clinical practice, cancer risk in A-T, and the use of MRI as a screening tool. | Outcomes relate to WB-MRI. Qualitative findings: Five themes emerged from the data: 1) cancer screening is "invaluable" - the belief earlier detection could improve cancer outcomes was seen as the most valuable aspect of screening, positive views about MRI not using ionising radiation and picking up non cancer-related issues as another advantage, also expressed; 2) cancer fears can increase anxiety - fear of cancer can increase anxiety, screening can add to this by reminding patients of A-T condition; 3) current practice for caring for people with A‐T puts pressure on families - lack of oncological support in particular to assist parents, as currently parents felt responsible for child's health; 4) effective preparation is essential for the cancer screening - provided with information and education about the purpose of the screening before the MRI scan, tailored to their age, experience, knowledge and background, and covering technical aspects associated with cancer surveillance (e.g. range of cancers that can be detected, the frequency of screening and the length of time to receive the results), help with coping with surveillance e.g. staff communication during the scan; 5) MRI screening presents challenges - noise, duration claustrophobia, need to lie still, option of anaesthesia to help but concerns about using this in children; provide local access to WB-MRI scans. |
| Omran et al., 2023 (Sweden) | Baseline and 1 year follow-up. Questionnaires completed before participants knew about results of WB-MRI. Measures comprised cancer worry (CWS), perceived benefits and barriers to surveillance participation, quality of life (SF-36). | Outcomes relate to WB-MRI. **Knowledge, attitudes and beliefs:** Perceived benefits were high and barriers low. All participants had a positive attitude towards WBMRI surveillance with no differences in perceived benefits (reduces my fear of cancer, provides me with a sense of control, gives me a sense of security, is a good way to detect cancer early) and barriers (can have a negative effect on my home mortgage and/or life and health insurance, can cause unnecessary worry, causes inconvenience in my life, screening is not important, if I am diagnosed with cancer it will be too late, is painful, is uncomfortable/ embarrassing), and these did not vary by personal history of cancer or change over time. **Psychological impact:** Cancer worry results presented for individual items, not aggregated scale. Cancer worry scores were higher among participants with personal history of cancer at the baseline scan on 7 out of the 8 items (p values ranged from 0.028 to <0.001). Changes in cancer worry did not differ according to previous cancer history, except on one item: thoughts about cancer risk affecting their ability to perform daily tasks decreased among people with a previous cancer history more than those without (p=0.014). 38% with a previous cancer history reported lower worry on this item while 95% of individuals with no previous cancer history reported no changes in worry. Compared with the normative age‐ and gender‐adjusted population data for SF‐36, the study participants had clinically significantly lower vitality and emotional role functioning items (role limitations due to mental health difficulties) (clinically significant=10 or more points difference). Participants with a previous personal cancer had significantly lower quality of life on some subscales at baseline: physical functioning (p=0.046), role limitations due to physical functioning (p=0.020), and general health (p=0.015), and social functioning (p=0.016) at one year follow‐up. |
| Que et al., 2025 (Singapore) | Cancer predisposition syndromes where WB-MRI recommended (Li-Fraumeni syndrome, Neurofibromatosis type 1, Von Hippel Lindau syndrome, Hereditary Paraganglioma-pheochromocytoma syndrome, Multiple Endocrine neoplasia Type 1, Fanconi Anaemia, Carney Complex). Measures: Patient charts were reviewed retrospectively to extract WB-MRI use, demographic details, genetic test results, cancer history, family history, and treatments received. | Outcomes relate to WB-MRI. **Uptake:** 57% uptake rate for WBMRI surveillance in those recommended to have it. Among those receiving WB-MRI, 37% had LFS, 24% had neurofibromatosis type 1, 14% with Von Hippel Lindau syndrome, and 14% with HPGL-PCC. |
| Rippinger et al., 2020 (Germany) | Measures: personal cancer history, familial cancer history, psychological support, logistical difficulties, satisfaction with genetic testing, whether would recommend genetic testing to family members, knowledge about and adherence to surveillance modalities and genetic counselling (intention to participate or active participation), cancer-related worry (CWS), perceived risk for cancer, and free-text comments about individual concerns and suggestions. | Outcomes relate to comprehensive screening including WB-MRI. **Knowledge, attitudes and beliefs:** Most individuals with LFS had general knowledge about recommended TP53 germline variants–specific surveillance (78%), 18% were unsure, and 4% were uninformed. Risk perceptions: A considerable proportion of the study participants (31%) underestimated their own cancer risk. Risk perception was not correlated with age, educational degree, employment status, a familial cancer history, or a personal cancer history. **Uptake:** Surveillance modalities were followed during the study or had been followed previously by 88%. 77% stated they had remained adherent to the surveillance after their LFS diagnosis, 23% were non-adherent (4% stopped participating, and 19% attended less often than recommended). Adherence was significantly more likely among people who expressed higher satisfaction with genetic testing and counselling (p=0.019) but was unrelated to demographics, cancer history, or level of TP53 germline variant–related distress. Reasons given for declined surveillance in the nonadherence group included; frequent confrontation with the condition, belief that further adherence would not be necessary after prophylactic mastectomy, and organizational problems. One-third of participants reported logistical difficulties in implementing surveillance due to high number of medical visits, scheduling difficulties, and commuting distance. Insurance companies covered the surveillance fully for 57% and partially for 12%. 31% of the participants could not provide any information on the coverage by their health insurance. **Psychological impact:** Almost half of participants reported being moderately (47%) burdened by thoughts of LFS with 20% reporting higher levels of burden. 57% experiencing strong to very strong worries for family members. 76% estimated that their partners were at least moderately emotionally burdened by their LFS status and 73% of family. Free-text comments were grouped into two main factors for distress (personal burden due to frequent follow-up visits and scheduling appointments a distance from home; and worries about their children’s health) and three factors related to improvements in practice (introduction of a standardized surveillance protocol and a central point of contact for the various diagnostic procedures; more information on their condition, including cancer prevention strategies and updates on new therapeutic options; and health insurance coverage for surveillance, including the costs for WB-MRI.) |
| Ross et al., 2017 (United States) | Questions focused on experience of LFS screening: emotional reactions to screening and test results, satisfaction and perceived efficacy of screening, drawbacks of the screening process, future screening intentions, and financial and logistical implications of screening. Participants took part in semi-structured interviews in person or by phone about their experiences with LFS screening. | Outcomes relate to comprehensive screening including WB-MRI. Qualitative findings: Themes were 1) perceived benefits: early detection, peace of mind, the value of centralised screening, and knowledge providing power, and screening making LFS seem more liveable; 2) perceived drawbacks: logistical issues (biggest one being insurance and whether it covered recommended screening either now or in the future and the worry arising from this), organisation and navigation within the programme (lack of organisation, communication errors, scheduling problems, lack of knowledge of the programme among other HCPs), feeling drained from screening (physically and emotionally, especially in relation to WB-MRI e.g. claustrophobia, stiffness due to scan duration), negative emotional reactions (fear, anxiety, scepticism about whether a "normal" screening result is correct). Half of the participants expressed experiencing fear related to screening, which was often related to claustrophobia during the MRI’s; another cause of fear was inconclusive or benign findings on the screening exams. 100% of patients plan to continue screening, with most citing loss of insurance coverage was the largest potential barrier to continue screening. |
| **Cancer Patients** |  |  |
| Adams et al., 2014 (The Netherlands) | Questionnaires completed on the day of scan, usually right after the scan. Measures: worry prior to undergoing scan, experience of the scan, feelings after the scan, experience of preparation for scan (CT only), and fear and pain during scan (paediatric patients only). | **Scan experience:** Patients reported significantly less worry before WB-MRI than before CT (1.47 vs. 1.83 scale 1-4, p=0.007), experienced WB-MRI as significantly less unpleasant (1.49 vs. 1.83, scale 1-5, p=0.01) and felt significantly better shortly after WB-MRI compared to CT (1.64 vs. 1.93, scale 1-4, p=0.003). Mean scores for worry and unpleasantness were low and scores for how they felt after the scan indicated high levels of wellbeing. Experience of preparation for CT (i.e. insertion of intravenous line, drinking of contrast fluid) was viewed as a burden. The necessary preparations before CT scanning (i.e. insertion of intravenous line, ingestion of contrast medium), are not required for whole-body MRI in staging of lymphoma, and were reported to be a considerable burden. |
| Dyrberg et al., 2018 (Denmark) | Questionnaires completed after all scans were done. Measures: prior experience of each scan, patient experience (overall and in relation to instructions from staff, confinement in scanner, intravenous injection, positioning of arms above head, fasting before scan, noise and heat), willingness to repeat scans, and most and least preferred scan assuming identical diagnostic performance. | Each patient had a NaF-PET/CT, a WB-MRI, and either a SPECT/CT (group A) or a choline-PET/CT (group B). **Scan experience:** Patients rated the experience of NaF-PET/CT as significantly better than WB-MRI (overall experience 2.0 vs 2.3, p value =0.0001; ratings for space in the scanner 1.5 vs. 1.8, p <0.0001) but there were no significant differences in scan experience between WB-MRI and either SPECT/CT or choline-PET/CT. Arm positioning was the most uncomfortable part of the three nuclear testing scans and acoustic noise was the most uncomfortable part of WB-MRI (stats in supplementary data not available). Previous scan experience and age did not influence overall experience ratings. Staff instruction was strongly correlated with overall scanning experience with all four techniques (r=0.36 to 0.49). **Scan acceptability:** Patients were willing to repeat all four imaging methods: NaF-PET/CT (100%), Choline-PET/CT (96%), WB-MRI (94%), SPECT/CT (90%). **Scan preference:** In group A there was a significant difference in scan preference (p<0.001) with NaF-PET/CT the most popular (32%), followed by no preference (29%), Choline-PET/CT (27%), WB-MRI (23%). There were no significant difference in scan preference in group B.  There were significant differences in the scan patients would most like to avoid in both groups (Group A, p=0.002; Group B, p<0.0001). Most patients in Group A preferred to avoid WB-MRI (approx. 47%). In Group B it was SPECT/CT (approx 35% - data shown in figure). |
| Evans et al., 2017 (United Kingdom) | Qualitative: questions on patient experience and acceptability of WBMRI in comparison with other scans (CT/PET-CT). | **Scan experience:** In general WB-MRI presented a greater challenge than PET-CT/CT due to enclosed space, noise and scan duration. Reduced patient tolerance was associated with claustrophobia, pulmonary symptoms and existing comorbidities. Coping strategies facilitated scan tolerance (e.g. distraction, breathing techniques, focusing on health benefit). Good staff communication helped reduce anxiety and physical discomfort. |
| Evans et al., 2018 (United Kingdom) | Baseline (some completed before the scan, some after): emotional distress (GHQ-12), self-reported comorbidities, demographics (age, gender, ethnicity, deprivation index). After the scan: scan burden (26-item scale: discomfort, worry, satisfaction), scan recovery, scan satisfaction, scan acceptability for both WB-MRI and CT/PET-CT. | **Scan experience:** The proportion reporting immediate recovery was not significantly different between scans (63.9% for WB-MRI vs. 65.1% for CT/PET-CT). Patients’ satisfaction with information before the scan and facilities, and communication during the scan were all high and not significantly different between WB-MRI and CT/PET-CT. Mean burden scores for WB-MRI were significantly greater than those of CT/PET-CT (2.21 vs. 1.81, p<0.001). The higher burden of WB-MRI was mainly due to items related to “discomfort”(2.51 vs. 1.83, p<0.001), WB-MRI conferred significantly greater feelings of claustrophobia and discomfort related to noise than CT/PET-CT: claustrophobia WB-MRI vs. CT (means scores 2.81 vs 1.51; p < 0.001) and WB-MRI vs. PET-CT (mean scores 3.04 vs 1.98; p < 0.001); noise: WB-MRI vs. CT (means 2.84 vs 1.73; p < 0.001) and WB-MRI vs. PET-CT (2.85 vs 1.63; p < 0.001). The intravenous injections required for each of the three scan types resulted in low levels of discomfort which did not differ between scan type. In unadjusted analysis, scan burden was higher in patients with comorbidities (β = 0.242, p = 0.015), higher psychological distress (β = 0.305, p = 0.002) and higher deprivation (β = −0.265, p = 0.005). Demographic variables and cancer type did not predict scan burden. In adjusted analyses, only psychological distress (β = 0.223; p = 0.025) and presence of comorbidities (β = 0.191, p = 0.048) remained significant predictors. **Scan acceptability:** Scan acceptability ratings were significantly lower for WB-MRI than CT/PET-CT (in lung patients 64.8% rated WB-MRI as very acceptable vs. 75% for PET-CT, p=0.035; in colorectal patients the figures were 65.5% vs. 80.4% for CT, p=0.005). |
| Mazzocco et al., 2023 (Italy) | Before the scan: anxiety (STAI-Y 1), personality (BIF-10); Revised Life Orientation Test (LOT-R); illness perceptions (IPQ-R). After the scan: anxiety (STAI-Y 1), scan preference (WB-MRI vs CT). | **Psychological impact:** There was no significant difference between pre-vs. post WBMRI anxiety scores, but participants had not received their post-scan results at this point. In adjusted analyses, higher pre-scan anxiety was significantly correlated with both higher emotional representation (β = 0.417, p = .003) and lower disease coherence scores (β = -310, p = .024) on the IPQ-R. Lower post-scan anxiety was significantly associated with higher dispositional optimism (β = - 0.600, p < .001. Demographic variables and cancer type were not significantly correlated with pre-scan anxiety. **Scan preference:** Out of 70, 55 preferred WB-MRI (78.6%), 5 preferred CT and 10 did not indicate a preference. |
| Miles et al., 2019 (United Kingdom) | Discrete choice experiment: attributes were time in a scanner, time to reach a final diagnosis, associated increase in cancer risk due to radiation, number of additional staging scans before final diagnosis, need for the head and whole body to be in a scanner. | **Scan preference:** Overall, participants preferred (i) to wait less time for a diagnosis, (ii) a lower dose of radiation exposure, (iii) fewer additional scans, and (iv) greater test accuracy. Lung cancer patients valued time to diagnosis most highly, followed by accuracy, radiation exposure, number of scans, and time in the scanner. Colorectal cancer patients valued accuracy most highly, followed by time to diagnosis, radiation exposure, and number of scans. There were no significant differences in preferences according to sub-groups stratified by gender, age, comorbidities, employment status, marital status, and positive mood. Thirty-seven percent of patients were ‘non-traders’ (participants whose preferences are determined by a single attribute, which they do not trade-off against any of the other attributes presented). The most common attributes patients would not trade were higher accuracy, faster time to diagnosis, and reduced cancer risk due to scan-related radiation exposure. Lung cancer patients were more likely to prefer a WB-MRI-based pathway (probability 0.64) if it was as accurate, required the same total number of scans, and had the same time to diagnosis as the default staging pathway. If the WB-MRI pathway was more accurate, reduced time to diagnosis and/or required fewer scans than the default staging pathway, then the preference for WB-MRI was even stronger. The same patterns were also found for colorectal cancer patients (who were more likely to prefer a WB-MRI-based pathway - probability 0.66 - if it was equivalent in accuracy, total scan number, and time to diagnosis compared with a standard staging pathway). When asked which they would prefer, 54.2% lung cancer patients and 73.8% colon cancer patients selected WB-MRI over standard scans. |
| Miles et al., 2020 (United Kingdom) | Baseline (some completed before some after the scan): comorbidities; emotional distress (GHQ-12); positive mood (PANAS); beliefs about WB-MRI (two items on whether ionising radiation used in WB-MRI or CT/PET-CT; three items on potential benefits of WB-MRI being tested in Streamline trials that had not yet been fully established: whether use of WB-MRI would lead to fewer scans in total for diagnosis and staging, might lead to a quicker or more accurate staging. After the scan: WB-MRI scan burden, preference for WB-MRI or CT/PET-CT | **Knowledge, attitudes and beliefs:** 85% of patients believed that WB-MRI might lead to more rapid diagnosis and staging, 75% that it might lead to fewer scans, and 49% that it might be more accurate than CT/PET-CT. 42% were aware that WB-MRI did not use ionizing radiation. 55% were aware that CT/PET-CT did use ionising radiation. CRC patients held significantly more positive beliefs about WB-MRI than lung patients (3.42 vs. 2.67, p = 0.003). When belief items were tested individually, only perceptions about accuracy were significantly different between CRC and lung cancer patients (66% vs 31% thought WB-MRI was more accurate than CT/PET-CT respectively, p<0.001). **Scan preference:** Overall, 52% expressed a preference for WB-MRI vs. CT/PET-CT. Preference for WB-MRI was significantly associated with higher positive mood scores (p=0.02); no comorbidities (p=0.005); beliefs in the potential benefits of WB-MRI (p=0.015); and lower perceived burden scores for WB-MRI (p=0.042). In adjusted analyses, only greater belief in benefits of WB-MRI remained a significant predictor of preference for WB-MRI (p=0.047). Examining the individual beliefs, only awareness that WB-MRI does not involve radiation predicted patient preference for WB-MRI (p=0.032): 64.4% of people who were aware WB-MRI did not use radiation preferred WB-MRI to standard scans while 43.5% of people unaware of this attribute preferred WB-MRI. |
| Oliveri et al., 2018 (Italy) | Before the scan: previous experiences of imaging examinations; pre-imaging anxiety and distress, concern about the procedure, patient's level of information, perceived utility for diagnosis, perceived risk related to the exam. After the scan: overall discomfort experienced, discomfort related to noise, feeling of confinement, shortness of breath, exam duration, level of perceived vulnerability, level of suffering during the exam, perceived scan utility, willingness to have again, scan satisfaction, acceptability in comparison with other diagnostic scans they have had. | **Knowledge, attitudes and beliefs:** Approximately two thirds of patients reported some degree of concern prior to the scan. 15.6% were very to extremely concerned, 43% slightly to moderately concerned. Pre-scan concern was higher for women than men (4.3 vs. 3.1, p<0.05) but was unrelated to age. The most common reason for concern was scan outcome (68%). 80.4% felt they received good information before the scan. 96.2% reported high or moderate levels of perceived scan utility (useful for diagnostic process). There was a significant association between higher perceived utility and higher perceived level of information provided before the scan (p=0.002). The majority (82.5%) did not perceive WB-MRI to be “risky”. **Scan experience:** A small percentage (3.9%) patients reported strong discomfort during the scan, 44.2% slight to moderate discomfort and 51.9% felt no discomfort. Noise and exam duration were the most common sources of discomfort, with the proportion reporting no discomfort lowest for noise (42.4%), followed by duration (46.6%), perceived vulnerability (50.4%), feeling of confinement (51%) and shortness of breath (66.4%). But the most common strong/extreme discomfort proportions related to feeling of confinement (10.7%) and perceived vulnerability (10.0%). There was a significant association between concerns prior to WB-MRI and discomfort experienced during the scan (r = 0.619; p < 0.001). Reported discomfort was not significantly related to age or gender. **Scan satisfaction:** Over 81% expressed high levels of satisfaction towards WBMRI, and higher satisfaction was significant associated with higher levels of information received before the scan (p = 0.002). **Scan acceptability:** 69% reported WB-MRI was more acceptable than other scans they had had (among those who had prior experience of MRI, CT or PET-CT). |
| Pinto et al., 2024 (Italy and Czech) | Measures: Patient experience/ satisfaction with each imaging modality (overall satisfaction, preparation before examination, duration of examination, noise during examination, enclosed space, pain during scan, experience adverse event). Concern about radiation exposure (CT only). Scan preference assuming equal accuracy. | **Knowledge attitudes and beliefs:** Fifty percent of the patients were not at all concerned about the radiation exposure of CT, 38% were a little concerned, and 9% and 3% of the patients reported that knowing about the radiation risk was moderately important or very important, respectively. **Scan experience:** Poorest experience in all domains was for WB-DWI/MRI – this was the imaging method with most “dissatisfied”/ “very dissatisfied” responses regarding preparation before examination (17% vs 11% CT and 1% ultrasound), duration of examination (30% vs 3% CT and 13% ultrasound), noise during examination (33% vs 4% CT and 1% ultrasound) and space comfort (36% vs 12% CT and 0% ultrasound). Patients reported significantly higher levels of pain during ultrasound than CT or WB-DWI/MRI - 78% reported no or mild pain, 19% moderate pain, 3% severe pain. There was no sig. difference in pain reported in CT vs WB-DWI/MRI, with 90% and 85% of patients reported no pain, respectively. Ultrasound was the preferred method regarding preparation before examination (proportion “very satisfied” were 66% for US, 48% for CT and 46% for WB-DWI/MRI) and space comfort (proportion “very satisfied” were 64% for US, 49% for CT and 28% for WB-DWI/MRI). Very satisfied with duration of exam: 49% US, 56% CT, 32% WB-DWI/MRI. **Scan satisfaction:** More patients were very satisfied with CT (56%) than US (51%) or WB-DWI/MRI (47%). **Scan preference:** All patients had all three scans and were asked which they would prefer if they were all equally accurate. 49% patients preferred ultrasound, followed by CT (38%), and WB-DWI/MRI (13%). None of eight tested variables was statistically significantly associated with scan preference (e.g. BMI, presence of symptoms, International Federation of Gynaecology and Obstetrics (FIGO) stage, or surgical outcome). |
| Quinn et al., 2024 (United Kingdom) | Measures: type of imaging received (MRI, CT, PET/CT, Skeletal Survey), number of imaging tests at first line imaging, and test combinations, time to imaging, A&E attendance, and presenting symptoms at A&E. | **Uptake:** There were differences in the type of first-line imaging patients received, but the effect size was small (r=0.11, p<0.001) so post-hoc comparisons were not computed. The majority of patients had more than one imaging test. A significantly larger proportion of patients received one or two scans (41.8% and 36.1% respectively) than three or four (18.3% and 3.8% respectively) at first-line imaging. MRI was the most common imaging received at diagnosis with 69.2% receiving this, followed by SS (48.7%), CT (43.1%), PET-CT (23.1%). The provision of first-line imaging in myeloma did not significantly change between 2016 to 2022, remaining unchanged since the guidelines were introduced. Imaging received did not differ by region (e.g. urban, rural) but did by geographical area (p<0.001): skeletal survey was used more often in East England (57.9%) and Scotland (61.2%) than South East England (36.3%) (p<0.05). |
| **Radiologists** |  |  |
| Feldhaus et al., 2020 (United States) | Measures: use of WB-MRI in the adult population, duration of use, indications for use, and sequences performed. | The survey was e-mailed to Society of Skeletal Radiology (SSR) members on September 19, 2018. 32% reported that their institutions routinely perform WB-MRI. 11% response rate. The majority of practices had offered WB-MRI for 5 years or less (68%). The most common indications for use were multiple myeloma (78%), tumour staging (58%) and cancer predisposition syndromes (32%). The most commonly performed sequences were coronal short tau inversion recovery (79%) and coronal T1 without fat saturation (73%). 48% respondents were not aware of the code used for billing WBMRI. The majority (59%) of WB-MRI exams were interpreted by a single MSK radiologist. Academic practices were significantly more likely to use WB-MRI (48%) than private (13%) or hybrid (24%), and the majority of institutions using it from more than 10 years were academic (80%). There was no significant relationship between type of practice and indications for performing WB-MRI, or most commonly performed sequences. |
| Westerland et al., 2018 (United Kingdom) | Measures: Their institution’s preferred first-line imaging for suspected myeloma, newly diagnosed myeloma, and solitary plasmacytoma; ranked preferences among SS, WB-CT, MRI spine, WB-MRI, and 18F-FDG PET/CT. Perceived barriers to implementing WB-MRI or PET/CT services. | Online survey of myeloma imaging practice between 01 September and 31 October 2017, endorsed by Myeloma UK, UK Myeloma Forum and the British Society of Skeletal Radiologists. This was publicised to their members via an online link. Participants comprised both clinical haematologists and radiologists with an interest in plasma cell malignancies. Skeletal survey was the most commonly performed first line imaging test for suspected or confirmed myeloma. Only 16.4% of responders offered WB-MRI. Only 9.8% of participants reported that WB-MRI was the preferred first-line imaging test at their institution for suspected and confirmed myeloma. Scanner capacity, dedicated reporting time, and lack of trained radiologists to report WBMRI were some of the commonly stated challenges. |
| Westerland et al., 2021 (United Kingdom) | Measures: routine availability of PET/CT, WBMRI, and WBCT; preferred first-line imaging techniques in suspected/newly diagnosed myeloma or plasmacytoma; preferred first-line imaging technique in smouldering (asymptomatic) myeloma; preferred first-line imaging technique in evaluating response to treatment; and barriers to implementing WBMRI, WBCT, and 18F-FDG PET/CT. | All UK radiology departments treating myeloma patients and with a Royal College of Radiologists (RCR) Audit Lead were invited to participate. Skeletal survey (SS) was available in most hospitals (88%). Onsite WB-MRI was available in 48% and offsite WB-MRI in 5%. In the absence of any imaging contraindications, SS was the preferred first-line imaging test in suspected/confirmed myeloma or plasmacytoma (39%), followed by onsite WBMRI (25%). Offsite WBMRI was performed first-line in 2% of hospitals. Where the first-line departmental imaging technique was contraindicated, the most frequently chosen alternative was SS (25%). For onsite WBMRI it was 11%, and offsite WB-MRI 1%. In smouldering myeloma, SS was the preferred imaging test in 36% of hospitals, followed by WBMRI (25%). SS was also the most popular first-line imaging test in response assessment (26%), despite NICE guidelines stating SS should not be used in patient monitoring. Onsite WBMRI was preferred first-line in response assessment (25% - 24% onsite, 1% off-site). Reported challenges to implementing a WBMRI service included scanner availability (52%), financial constraints (49%), reporting time (46%), and radiologist training (46%). 40% of hospitals stated that they would be happy to provide support to other departments considering setting up a WBMRI or WBCT service. |
